# New 28-Item and 12-Item Dog Owner Relationship Scales: Contemporary Versions of the MDORS with a Revised Four-Component Structure

**DOI:** 10.3390/ani15050632

**Published:** 2025-02-21

**Authors:** Pauleen C. Bennett, Deanna L. Tepper, Louisa Rogers, Chiara Mariti, Tiffani J. Howell

**Affiliations:** 1 Anthrozoology Research Group, School of Psychology and Public Health, La Trobe University, Bendigo, VIC 3552, Australia; d.tepper@latrobe.edu.au (D.L.T.); t.howell@latrobe.edu.au (T.J.H.); 2 Department of Veterinary Sciences, University of Pisa, Viale delle Piagge, 2, 56124 Pisa, Italy; chiara.mariti@unipi.it

**Keywords:** Dog–Owner Relationship Scale, Cat–Owner Relationship Scale, social exchange theory, Lexington Attachment to Pets Scale, pet–owner relationships

## Abstract

Numerous scales have been developed to assess pet–owner relationship quality. One commonly used measure is the Monash Dog Owner Relationship Scale (MDORS) and its various derivatives. Since this scale was published in 2006, many social changes have occurred, necessitating a review and, if necessary, refinement of this measure. We sought to investigate the internal consistency and structure of the existing instrument, as well as an expanded and modified version of the scale, in a contemporary adult sample of over 350 adults, recruited to be potentially less dog-centric than previous samples. The existing three-factor structure of the MDORS appeared reasonably sound, but a Principal Components Analysis with modified items resulted in identification of a four-component structure. Two of the components approximated existing MDORS measures: the Perceived Costs of Dog Ownership and the owner’s Emotional Reliance on their pet. Rather than replicating the existing Pet Owner Interaction subscale, however, we identified two different types of engagement: Affectionate Engagement and Active Engagement. The subscale scores and the total score were, as expected, intercorrelated with each other and with the Lexington Attachment to Pets Scale. Perhaps surprisingly, however, they were statistically associated with very few demographic variables, such as owner gender, age, education, or residence location, and they were rarely associated with dog variables such as age, sex, or source. This may speak to the current near-universality of strong human–dog relationships, which we propose can now be assessed using two new measures: the Dog Owner Relationship Scale 28 (DORS28) and a shortened version, the Dog Owner Relationship Scale 12 (DORS12).

## Simple Summary

Over the past two decades, the human–dog relationship has changed significantly, necessitating updates to existing measures of this bond. The aim of this study was to review and, if necessary, refine the Monash Dog Owner Relationship Scale. Although the MDORS was found to be reasonably robust, minor modifications resulted in a four-component structure being identified, reflecting Perceived Costs of Dog Ownership, Affectionate Engagement, Emotional Reliance, and Active Engagement. Two new questionnaires were then developed: the Dog Owner Relationship Scale 28 (DORS28) and its shortened version, the Dog Owner Relationship Scale 12 (DORS12). Scores on both questionnaires were associated with few owner or dog characteristics, perhaps demonstrating that contemporary dog–owner relationships are almost universally perceived by owners to be very strong.

## 1. Introduction

Accurately measuring the relationship between a person and their companion animal (hereafter referred to as “pet”) is important for those seeking to understand, among other things, whether specific animal and human characteristics are more conducive to good relationships than others and if interventions designed to improve human–pet relationships are successful. This has led to many attempts to measure the strength and characteristics of human–pet relationships in a variety of different ways [1]. Particularly popular among researchers are written surveys. These are relatively quick to administer and can easily be incorporated into many experimental designs, although a limitation is that they are typically one-sided, measuring the human’s perception of the relationship rather than the animal’s perception. Notwithstanding this limitation, there have been many attempts to develop scales to quantify the quality or nature of human–pet relationships (see [2] for a review).

Scales of this kind typically focus on the benefits of pet ownership and/or how close an owner feels to their pet [3,4]. While people do typically report experiencing many benefits associated with pet ownership [5], there are also financial, time, and emotional costs involved in having a pet. Financial costs can be unexpectedly high, particularly if animals develop serious medical issues or require boarding while an owner travels or is unwell [6]. Owners may also forgo experiences that they would ordinarily enjoy due to the need to care for their pet, and loss of a pet can take an emotional toll [7,8]. Animals, particularly dogs, that behave in ways that are undesirable can result in many adverse effects for owners, including poor mental health outcomes, impaired social relationships with other people, and an increased burden of responsibility [9].

Initially proposed by George Homans in the 1950s [10], social exchange theory holds that people consider the potential costs and benefits of social relationships, choosing to maintain specific relationships only when the benefits outweigh the costs. This theory was used to inform development of the Monash Dog–owner Relationship Scale (MDORS) in 2006 [11]. The influence of this scale within the field of anthrozoology has been substantial. A Google Scholar search, conducted in November 2024, indicated that the paper reporting development of the MDORS has been cited more than 250 times, with hundreds more publications citing studies that used the scale. The MDORS has also been translated into several languages [12,13,14] and modified for use in species other than dogs [15,16].

The MDORS continues to be widely used, suggesting that a review of the scale is appropriate. Pet ownership has changed quite markedly since its publication in 2006; indeed, the very concept of ownership is now controversial [17]. Dozens of studies have confirmed that human–dog relationships can be, but are not always, beneficial for humans (see [5] for a review) and, also, that dogs are able to develop strong affectionate bonds with humans [18,19]. Public interest in these studies has led to widespread dissemination, including via general interest books authored by respected scientists [20,21,22], and may have resulted in a corresponding change in how dogs are perceived. Widespread social media use has led to a rise in pet-promoting influencers, many of whom showcase quite intimate relationships with their animals, while the COVID-19 pandemic led many people to rely heavily on their pets for social and emotional support [23,24]. It might be anticipated, therefore, that emotional closeness may have been replaced with emotional reliance on pets for some owners. There has also been a pronounced increase in the popularity of low-shedding, curly-coated, crossbred dogs [25], and this may have altered the ways in which some owners interact with their pets. Many of today’s most popular breeds are small and have high healthcare and grooming needs, perhaps resulting in more grooming, hugging and petting, and less engagement in active dog sports. Changes in the costs associated with dog ownership have also occurred, with increased spending on numerous products and services, proposed to enrich pets’ lives, being well documented [26].

In addition, as researchers, we have long been concerned by some aspects of the MDORS. While the MDORS was comprised of three components: Pet Owner Interactions (POIs), Perceived Emotional Closeness (PEC), and Perceived Costs (PCO), these were not validated with a Principal Components Analysis in the original publication [11]. Also, research has demonstrated repeatedly that many items in the MDORS have limited variability, being strongly skewed towards more positive perceptions of the relationship. This likely reflects the true nature and depth of modern relationships with companion dogs. We also believe, however, that contemporary studies are affected by a common recruitment bias, being dependent on attracting voluntary participants, many of whom are so invested in their dogs that they regularly engage with the dog-focused social media sites used to recruit participants. This is unavoidable and, to some extent, targets the participants in whom researchers are often interested. It may, however, provide an unrealistically positive view of human–dog relationships in the general community.

Aside from these conceptual issues, we also feel that the choice of response options in the MDORS was sometimes poor. For example, two items are ‘How often do you give your dog food treats?’ and ‘How often do you hug your dog?’ In the original scale, the available response options for these items are ‘At least once a day’, ‘Once every few days’, ‘Once a week’, ‘Once a month’, and ‘Never’. It is inappropriate to assume that these options are anything other than ordinal level, making it difficult to justify the application of many common statistical procedures. It is also fairly evident that responses are likely to strongly cluster around the first and last options. Informal observations suggest that some people are philosophically opposed to feeding dogs food treats or hugging their animals. Those who do engage in these activities, however, appear to do so very frequently.

Riggio et al. [27], in the Italian version of the MDORS, broadened the response options from five to seven. They also reversed the response options, so that the rightmost options indicated the most frequent occurrence of the behavior. Whilst we agree that these innovations improve the MDORS, the problem of assuming the data are interval level for statistical purposes remains; the interval between ‘Once a month or less’ and ‘About once a week’ is not equivalent to the interval between ‘More than once a day’ and ‘About once an hour’. Engaging in some of the behaviors is also constrained by availability; while a person may wish to hug their dog many times per day, this might not be possible if they spend little time at home. Although objective response options are generally preferred and less vulnerable to socially desirable responding, evaluating more subjective options appears justified in this situation. In fact, many MDORS items already have subjective response options, ranging from ‘Strongly disagree’ through to ‘Strongly agree’. Similarly, knowing whether a dog is hugged once per week or many times per hour may reveal less about the perceived strength of a relationship, from the person’s subjective point of view, than knowing whether they feel their dog is hugged ‘infrequently’ or ‘extremely often’. A final limitation of the MDORS is that, with 28 items, it is quite long. Many requests for a shorter form have been received by the original authors but, to our knowledge, no short form has yet been published.

The first aim of the current study was to assess the internal consistency and component structure of the MDORS, using seven-point rather than five-point response options, in a sample selected to be more representative of the general population than many previous studies have been able to obtain. A second aim was to attempt to address some of the shortcomings of the MDORS. This was achieved by expanding the item pool by the addition of four extra items and replacing objective response options for many items with subjective options. We then conducted a series of Principal Components Analyses, seeking to identify a structure that best fit the data. Finally, we explored whether a shortened form of the modified scale could be developed.

## 2. Materials and Methods

This study collected anonymous, non-sensitive data from over 350 adult volunteers capable of providing informed consent. Consequently, it was exempted from requiring approval by the La Trobe University Human Ethics Committee (Exemption Number: EIBX24011). The sample was recruited from the USA using Prolific Academic, an online platform where registered users participate in research on any topic in exchange for small payments. Using filters provided by Prolific (https://www.prolific.com; accessed from 1 October 2024–18 October 2024), only site users who were at least 18 years of age, a dog owner, a resident of the USA, and able to read and write in English were invited to participate. Over 19,000 eligible participants were registered on the Prolific site.

### 2.1. Participants

In total, 373 eligible Prolific users commenced the survey. After removing data from those who failed to provide a full data set, 354 participants remained. Demographic information about the participants is summarized in Table 1. People who identify as females were slightly over-represented. Most participants were relatively well educated. Nearly half lived in a suburban location. Most lived in a house. Few lived in a residence with no outside space. Over half the sample had full-time paid work. Most participants lived with one other adult or in single adult homes. Most lived with no children.

Information about participants’ dogs is presented in Table 2. Almost two-thirds of the participants owned just one dog, but many also owned at least one cat or some other kind of pet. Most had first lived with a dog as a child, and nearly all had lived with at least one dog previously. The age of the dogs owned by participants varied widely, but most had been acquired as puppies. Male dogs were more common than female dogs, and dogs of both sexes were mostly spayed or neutered. Dogs were acquired from a wide range of sources. The majority spent their time mostly inside the house, with very few spending most of their time outside. Participants engaged in a range of activities with their dogs; very few did not engage in any activities. Nearly all participants were satisfied or very satisfied with their dog’s behavior and health. Participants mostly felt that the quality of their relationship with their dog was much better or somewhat better than for other dog owners, with very few feeling that their relationship was either somewhat worse or slightly worse than others.

### 2.2. Materials

An online survey, consisting of five sections, was created using REDCap (projectredcap.org), a secure online data capture application. A full copy of this survey is available in the (Supplementary Materials File S1: Survey).

#### 2.2.1. Owner and Dog Demographics

The first section asked about participant demographics (e.g., gender, year of birth, employment status, education level, and household composition and location). The second began by asking the participant to provide their pet dog’s name. Participants who owned more than one dog were asked to complete the survey about whichever of their dogs’ names started with the letter closest to ‘A’. The name provided was used to automatically personalize the remainder of the survey, with [petname] being replaced by the reported name in each question. For example, a participant who reported that their dog’s name was Max was asked ‘How old was Max when you got him/her?’ and ‘How often do you buy Max gifts?’. The remainder of this section comprised questions related to dog demographics (e.g., current age, age at acquisition, source of acquisition, sex, and weight). It also contained general questions about the owner’s level of satisfaction with the dog’s behavior and health and the types of activities they engaged in with their dog (e.g., tracking, obedience training, and animal-assisted interventions).

#### 2.2.2. MDORS with Updated Items and Varying Response Options

The third section was a 49-item scale that we developed and called the Expanded Dog Owner Relationship Scale (E-DORS). This included all 28 items from the original MDORS [11], with just a few minor changes to wording where this was necessary to clarify items or assist interpretation. It also included four additional items from the C/DORS [16]. These four items were identified by Howell et al. [16] as being particularly informative regarding cat–owner relationships and were not previously incorporated into subscale scores for dog owners. They were included in this study to capture data about how frequently owners demonstrated affection towards their dog by engaging in activities like kissing, cuddling, petting, and watching their pet.

The response options for all items were increased from five to seven points, presented as Likert-type scales, and all were arranged so that the rightmost option represented the highest level or amount of the concept in question, as per Riggio et al. [27]. For example, for the item ‘How difficult is it to look after [petname]?’, the response options ranged from 1 = ‘Very Easy’ to 7 = ‘Very Difficult’.

Seventeen of the thirty-two items (13/28 MDORS items and 4/4 C/DORS items), which originally had objective response options, were presented twice, once with the original objective (O) response options and once with revised, more subjective (S) response options. The second presentation was randomly dispersed throughout the existing scale but in identical order for each participant (see Supplementary Materials File S1: Survey). For example, for the item ‘How often do you kiss [petname]’, the objective response options in Question 5 ranged from 1 = ‘Once a month or less’ to 7 = ‘More than once an hour’. In the revised, subjective version of this item (Question 11), these options were replaced with ‘Never’, ‘Almost never’, ‘Occasionally’, ‘More than occasionally’, ‘Often’, ‘Very often’, and ‘Extremely often’.

#### 2.2.3. Lexington Attachment to Pets Scale

The fourth section of the survey contained the Lexington Attachment to Pets Scale, or LAPS [4], a commonly used measure of pet–owner relationships with good validity and reliability. This 23-item scale has three dimensions: general attachment, people substituting, and animal rights/animal welfare. Items are scored on a four-point Likert-type scale, with a higher score indicating a stronger level of agreement with that item. In the current sample, the total LAPS had a Cronbach’s α of 0.859 (M = 38.5; SD = 6.10). Cronbach’s α results for the subscales were 0.76 (general attachment), 0.79 (people substituting), and 0.87 (animal rights/welfare).

#### 2.2.4. Final Questions About the Dog–Owner Relationship

The LAPS was followed by three additional questions in the fifth section of the survey, asking participants how bonded they believed their dog was to them on a seven-point scale, where 1 = ‘Not at all’ and 7 = ‘Extremely’. Using free text, participants were also asked to provide three adjectives that best represent their relationship with their dog and three adjectives that best represent their dog. These free text responses are not reported in this publication.

### 2.3. Procedure

Data collection commenced at 9 p.m. on 14 October 2024 US Eastern Daylight Time (EDT; 6 p.m. Pacific) and was completed 24 h later. Throughout this period, the survey was activated regularly for short periods to encourage participation from different time zones across the USA and from people with a variety of daily schedules. Eligible participants received a brief description of the study and an invitation to participate via Prolific. Those who responded were directed to the Participant Information and Consent Form, hosted by REDCap. This provided a more detailed description of the study, including advice that many of the questions might be similar or even identical, so that participants should review response options carefully before responding. Participants who consented to continue were then directed to the survey. The median time for survey completion was 12:47 min, for which participants received a small payment of GBP 1.29.

### 2.4. Analysis

Data were exported from REDCap to Microsoft Excel (version 2411) for cleaning, with incomplete responses deleted before the data were transferred to Jamovi statistical software (version 2.3) and IBM SPSS Statistics for Windows (Version 29.0.2.0) for analysis. Descriptive statistics were obtained, subscales calculated, and assumptions tested as appropriate for each analysis. The Principal Components Analyses (PCA) described below, for example, were employed only after examination of Bartlett’s test of sphericity and the Kaiser–Meyer–Olkin Measure of Sampling Adequacy, which, in all cases, were excellent. All PCAs were conducted using SPSS, with parallel analyses being obtained using Monte Carlo PCA for Parallel Analysis, ©2000 by Marley W. Watkins. Jamovi was used to calculate subscale scores, generate descriptive statistics and correlations, and to test hypotheses about group differences.

#### 2.4.1. Data Cleaning and Descriptive Analyses

The 49 items from the E-DORS were processed as follows. First, some items were reverse-scored (RS), so that higher scores always represented what we consider to be a ‘stronger’ human–companion dog relationship. These items are identified in Table 3. Second, a full range of descriptive statistics was obtained for each item, including items that were presented twice, once with the original O response options and once with revised S response options. These are also identified in Table 3. As is evident from this table, increasing the number of response options from five to seven was partially successful in terms of increasing variability, with the full range of options, from 1 to 7, being selected for all questions except two, E-DORS Items 16 and 19. For these two items, responses ranged from 2 to 7. It also appeared that rewording the options from objective to subjective terms often, but not always, improved the skewness and kurtosis of the items. Many items, nonetheless, obtained very high skewness and kurtosis values, with statistically significant Shapiro–Wilk’s tests of normality for all variables confirming that none were normally distributed. Tabachnick and Fidell [28] warned against placing too much emphasis on skewness and kurtosis values in large (200+) samples, but observation of histograms and Q–Q plots (not presented) visually confirmed the marked non-normality of many items.

#### 2.4.2. Assessment of the Original MDORS

To address the first aim of the study, an internal consistency analysis was conducted for the 28 items extracted from the MDORS, using the original objective response options for items administered twice and, for all items, the expanded seven-point item scales. Although the MDORS was initially considered to comprise three distinct subscales, some authors (e.g., [12]) generate a score for the total scale to represent a global measure of dog–owner relationship quality. For this reason, the internal consistency of the entire scale was examined, as well as the internal consistency of the subscales.

A PCA was conducted using the same 28 items. This method of analysis was selected over a confirmatory factor analysis, so that the results were not constrained by the previous model [29], which was not validated statistically in the original publication. Maximum Likelihood was selected as the extraction method and Direct Oblimin rotation applied because the components were expected to be correlated. A Parallel Analysis was used to inform how many components to extract.

This analysis generated a three-component solution, with four items cross-loading on two components. These items were therefore removed and the analysis repeated. Additional analyses were also conducted after removing items with very high or high skewness and kurtosis values and altering extraction and rotation methods. The outcomes for only the initial analyses are reported in this publication, as none of these strategies resulted in a more acceptable solution.

#### 2.4.3. Assessment of the Expanded and Modified E-DORS

The four items extracted from the C/DORS were added to the MDORS data set. All items with objective response options were removed, so that only the identical items with subjective response options were retained. These 32 items (28 from the MDORS and 4 from the C/DORS), all with seven-point subjective response options, were then entered into a PCA using the Maximum Likelihood extraction method, Parallel Analysis, and Direct Oblimin rotation. PCA was applied to assist with generating an empirical summary of the data [28]. To be as inclusive as possible, items were retained if they loaded on any component at greater than 0.3. If they cross-loaded at greater than 0.3 on two or more components, they were removed from subsequent analyses. Once an optimal model was identified, Cronbach’s α was used to assess the internal consistency of the components and the total scale. The highest loading items from each component were then investigated further using another PCA and Cronbach’s α to establish whether a statistically acceptable short form of the scale could be identified.

#### 2.4.4. Subgroup Differences and Correlations Between DORS Scales and Other Variables

At the conclusion of these analyses, a four-component model of the data was selected for retention, comprising 28 items. A 12-item short form of this same model was also retained. The components of these models were compared with each other, the LAPS, and other survey items to begin to determine the convergent validity. Associations with relevant demographic variables were determined in the final stages of analysis, using a combination of correlations and analysis of variance, or their nonparametric alternatives, as appropriate.

## 3. Results

### 3.1. Analysis of the Original MDORS

Selected results for this section are presented in detail in the (Supplementary Materials File S2: Analysis of the original 28-item MDORS). The main outcomes can be summarized as follows.

The internal consistency analysis of the 28 items making up the original MDORS, but with response options ranging from one to seven rather than one to five, confirmed that the original scale is internally consistent (File S2, Analysis A). The Cronbach’s α for each of the three subscales exceeded 0.83 and, for the entire 28-item MDORS, was 0.91. Although the data required to directly compare 5-point with 7-point scales were not collected, the means for two of the subscales were above 5 on the 7-point scales. This suggests that increasing the range was beneficial.

Visual examination of correlations between MDORS items suggested that the data were suitable for PCA. Many of the almost 500 correlations were above 0.3 and none exceeded 0.8, although three correlations exceeded 0.7.

PCA of the existing 28 MDORS items revealed six components with eigenvalues exceeding 1. Visual examination of the scree plot and a parallel analysis, however, suggested that only three components should be retained (File S2, Analysis B). Repeating the analysis with the number of components fixed at three (File S2, Analysis C) resulted in four items cross-loading at >0.3 (MDORS15, MDORS16, MDORS18, and MDORS22). When these items were removed and the analyses repeated (File S2, Analysis D), the three-component solution revealed a simple structure, accounting for 53.97% of the variance. The three components were broadly comparable to the subscales currently extracted from the MDORS.

The first component, accounting for 35.39% of the variance, contained ten items, all loading at greater than 0.42. Nine of these are typically included in the Perceived Emotional Closeness subscale of the MDORS (MDORS2, MDORS5, MDORS13, MDORS19, MDORS21, MDORS23, MDORS25, MDORS27, and MDORS28), which also includes MDORS15, one of the items eliminated due to cross-loading. The tenth item included in the first component (MDORS26; How often do you have [Petname] with you while relaxing, i.e., watching TV?) is currently included in the Pet Owner Interaction subscale of the MDORS.

The second component contained six items, all of which are currently included in the Perceived Costs subscale of the MDORS. All loaded negatively on this component at >0.51. In the MDORS, this subscale also includes three of the four items eliminated due to cross-loading (MDORS16, MDORS18, and MDORS22). Factor 2 explained 11.48% of the variance and was moderately negatively correlated with Factor 1 at −0.472

The third component contained eight items, all of which are currently included in the Pet Owner Interaction subscale of the MDORS. Missing from this component was MDORS26, which, as described above, loaded on Component 1 rather than Component 3. All items retained in the third component loaded at >0.395. This component accounted for 7.095% of the variance and was moderately positively correlated with Component 1 (0.521) and weakly negatively correlated with Component 2 (−0.206).

These analyses together confirmed that the subscales and total score for the MDORS are reasonably robust and internally consistent, as has been reported in numerous previous publications from several different countries. With the exception of one item, which loaded on a different component than expected, and four items that were removed due to cross-loading on two components, each item grouped with other items as expected, essentially replicating the existing structure of the MDORS almost two decades after its original development.

### 3.2. Analysis of the Expanded and Modified E-DORS

For the 17 items where both objective and subjective response options were trialed, the O items were discarded. After confirming that the data were suitable for PCA, with numerous correlations exceeding 0.3 but no correlations exceeding 0.8 (9 exceeded 0.7), all 32 remaining E-DORS items (28 from the original MDORS and 4 from the C/DORS) were entered into a PCA (see the (Supplementary Materials File S3: Analysis of the expanded and modified 32-item E-DORS)) with the Maximum Likelihood extraction method and Direct Oblimin rotation. This revealed five components with eigenvalues exceeding one, although the scree plot was more indicative of three components and a Parallel Analysis suggested that four should be retained (File S3, Analysis A).

Based on the Parallel Analysis, a four-component solution was extracted when this analysis was repeated. This resulted in a solution accounting for 58.16% of the variance but with four items (MDORS2, MDORS5, MDORS19, and MDORS26) cross-loaded at 0.3 (File S3, Analysis B). These items were removed and the analysis rerun, after confirming with a Parallel Analysis that four components remained appropriate with the number of items remaining. This resulted in a four-component solution accounting for 58.98% of the variance and with a simple structure (File S3, Analysis C).

As is evident from Table 4 and Table 5, the four components included in this 28-item solution appear quite distinct and conceptually meaningful. Component 1 contained nine items. Five were drawn from the Pet Owner Interaction subscale of the MDORS. These were joined by all four of the new items added by Howell et al. [16]. Cronbach’s α for this component was 0.886, and the lowest loading was 0.328. It appeared to mostly reflect person–pet interactions, which act to demonstrate the person’s affection for their animal, containing items such as ‘How often do you kiss [Petname]?’ and ‘How often do you hug [Petname]?’. Factor 1 was named ‘Affectionate Engagement’ (AFF).

Component 2 contained all nine items drawn from the Perceived Costs subscale of the MDORS (for example, ‘It bothers me that [Petname] stops me doing things I enjoyed before I owned him/her.), so this name was retained (Perceived Costs; PCO). All items loaded at >0.50, and the Cronbach’s α for this component was 0.887.

Component 3 (Cronbach’s α = 0.892) included five items, all drawn from the Perceived Emotional Closeness subscale of the MDORS. The items in this component all loaded strongly (>0.558), and they consistently conveyed a sense of the participant being emotionally dependent or reliant on their dog. Items included ‘[Petname] is there whenever I need to be comforted’ and ‘[Petname] helps me get through tough times’, so we named this third component ‘Emotional Reliance’ (EMR).

Five items were also included in Component 4. These items also loaded quite strongly (>0.348) and were internally consistent, with a Cronbach’s α of 0.781. All five items were drawn from the Pet Owner Interaction subscale of the MDORS, and they appeared to reflect relatively active person–pet engagement. For example, ‘How often do you take [Petname] to visit people?’ and ‘How often to you play games with [Petname]?’. Thus, we named this fourth component ‘Active Engagement’ (ENG). In addition to all four subscales achieving a Cronbach’s α in the range of 0.78 to 0.89, the overall scale was internally consistent, with the Cronbach’s α being 0.923 (see Table 5).

### 3.3. Generation of a Short Form

To evaluate the possibility of creating a brief instrument with which to assess the four components identified above, we entered into a PCA the two highest loading items from each of the four components in the 28-item solution. This produced an unsatisfactory result, with the items grouping into just two components with eigenvalues exceeding one and two items attaining loadings exceeding one. We therefore repeated this analysis with the three highest loadings from each component (File S3, Analysis D). This resulted in a solution with four components with eigenvalues exceeding one, which accounted for 75.90% of the variance, and had all 12 items grouping as expected, with no cross-loading. While a Parallel Analysis suggested that three components might better accommodate the data, the Cronbach’s α exceeded 0.74 for each of the four three-item subscales and was 0.838 for the total 12-item scale (see Table 5). It was therefore decided that these 12 items could be used as a reasonable approximation of the longer scale.

### 3.4. Characteristics of the Preferred Models

The components identified in the 28-item and 12-item solutions were used to generate subscale scores. This was done by adding the relevant item scores and dividing by the number of items included, so that the possible range for each subscale was 1–7. Total scale scores were also calculated by summing the four relevant subscale scores, so the possible range for these, in both instruments, was 4–28. Total scores for both the 12-item and 28-item scales, while not normally distributed, were within acceptable skewness and kurtosis ranges (Table 5). Individual participant scores were distributed across the entire range for most measures, although skewness was lower than -1 for two subscales in the 28-item model and for the corresponding two subscales in the 12-item model. Skewness was within an acceptable range for all other measures. Kurtosis exceeded +2 for the EMR subscale in both measures but was adequate in all others. The means exceeded 6 in the 7-point scales for both EMR subscales and were close to the mid-point for both ENG subscales. On all other measures, they were above the mid-point of the possible range but also well below the possible ceiling. This suggests that, while some individual item scores are heavily skewed (Table 3), they can, with caution, be combined into summary measures with properties able to support standard statistical approaches. Histograms and descriptive statistics for all summary measures are provided in File S4: DORS28 and DORS12 subscale properties and relationships with other variables.

The validity of the two new instruments was partially examined by generating relevant correlations (Table 6 and File S4: DORS28 and DORS12 subscale properties and relationships with other variables). Spearman’s rho was used rather than Pearson’s to account for variables that were ordinal and, typically, not normally distributed. This showed, first, that, within each measure, the subscale scores were typically moderately to highly intercorrelated and, second, that the short form of the scale may be a reasonably good approximation of the longer form. Correlations between DORS28 subscales and the corresponding DORS12 subscales always exceeded 0.883, with DORS12-TOT correlating with DORS28-TOT at 0.952. The subscales within the two measures were also robustly positively intercorrelated. Only two intercorrelations were not significant at *p* < 0.01 (DORS12-PCO/DORS28-ENG (*p* = 0.017) and DORS12-PCO/DORS12-ENG (*p* = 0.083)), suggesting that the Perceived Costs of Ownership, at least in the short form of the scale, may not vary systematically, depending on the degree of Active Engagement between owner and dog.

Correlations were calculated between the DORS measures and LAPS subscale and total scores (Table 6). For the DORS28, these were always positive, moderate to strong, and significant at *p* < 0.001. The correlation between the LAPS total score and the DORS28 total score was 0.782. Correlations between LAPS measures and DORS12 measures were also always positive, moderate to strong, and significant. As would be expected in a short form, they tended to be slightly less robust than for the DORS28; for example, the correlation between the total score for the LAPS and the total score for the DORS12 was 0.714.

Owner satisfaction with their dog’s behavior and health, the degree to which they thought their relationship with their dog was of a higher quality than other peoples’, and the degree to which they felt their dog was bonded to them were positively correlated with all measures extracted from the DORSS28 and DORS12. Most correlations were moderate to strong, although some were weaker; participants’ satisfaction with their dog’s health was only weakly positively correlated (Spearman’s rho from 0.11 to 0.219) with all the subscales. This may indicate that dog owners’ relationship with their dog is relatively unaffected by the dog’s health status.

Correlations were also calculated for the DORS28 summary measures and numerous demographic variables from the data set (Table 6). Perhaps surprisingly, very few were significant. Participant age was weakly positively correlated with DORS28-PCO but not with any other measure. Education level was weakly correlated with DORS28-ENG and, more weakly still, with DORS28-TOT. The number of adults in the home was weakly negatively correlated with DORS28-EMR and DORS28-TOT. The number of children in the home was also weakly negatively correlated with these two measures and with DORS28-PCO and DORS28-EMR. The number of dogs currently living in the home was weakly positively correlated with DORS28-PCO and weakly negatively correlated with DORS28-ENG. Dog age was also negatively correlated with DORS28-ENG, as well as being weakly positively correlated with DORS28-PCO. The age at which the dog was acquired was not associated with any DORS measures. Neither, perhaps surprisingly, was the size of the dog, operationalized by asking owners to estimate their dog’s current weight.

Group differences on the DORS28 measures were tested using analysis of variance techniques or appropriate non-parametric alternatives. These analyses are summarized only briefly here, with full details available in File S4: DORS28 and DORS12 subscale properties and relationships with other variables. A general lack of significant differences was the most interesting finding to emerge from the numerous analyses conducted.

Total scores on the DORS28 did not differ significantly across participant gender. Subscale scores were also consistent across males and females, the only exception being for DORS28-AFF, upon which females scored significantly more highly than males. Other genders were too infrequent to analyze. The DORS28-ENG scores varied significantly by employment status, although the only significant post-hoc comparisons were between participants employed full-time and those either unable to work or occupied with home duties and also between those employed part-time and those unable to work. Kruskal–Wallis tests also revealed significant differences across marital status on three measures: DORS28-PCO, DORS28-EMR, and DORS28-TOT. Very few post hoc comparisons were significant, but those that were indicated that married participants sometimes obtained slightly lower scores than participants who described themselves as separated, divorced, or widowed. DORS28-ENG was the only variable that varied significantly with whether participants lived in a suburban, rural, or regional location, but no post hoc comparisons were significant. There were no differences across the type of accommodation participants lived in and no differences across residents whose accommodation had more vs. less outside space, although DORS28-ENG and DORS28-TOT did vary depending on where the dog spent most of its time. Post hoc tests revealed higher DORS28-ENG scores for dogs with both indoor and outdoor access and lower DORS28-TOT scores for dogs kept primarily outdoors, but these results should be interpreted with caution given the very low number of dogs (12) in this category compared to the high number kept primarily inside (251) or with equal access to indoors and outdoors (91).

There were no significant differences on DORS measures across four dog sex groups (male desexed, female desexed, male intact, and female intact) or dog source, but there were some significant differences depending on whether participants engaged in specific activities with their dog, such as engagement in dog sports (DORS28-AFF, DORS28-ENG, and DORS28-TOT); obedience training (DORS28-AFF, DORS28-ENG, and DORS28-TOT); and trick training (DORS28-AFF, DORS28-EMR, DORS28-ENG, and DORS28-TOT). In all cases, participants who reported engaging in these activities obtained higher scores on the relevant subscales than those who did not engage. To some extent, this likely reflects overlap in these items and some of the items in the DORS measures, but it will be interesting in future work to verify these findings. Engagement in activities may be a way to promote stronger owner–dog relationships following the adoption of dogs from shelters.

## 4. Discussion

The first aim of this study was to examine the internal consistency and structure of the Monash Dog Owner Relationship Scale (MDORS) [11], an existing measure of human–companion dog relationships, in a contemporary USA adult sample recruited through Prolific. It was anticipated that this recruitment strategy would result in less dog-centric participants and more variability than many previous studies using the measure have been able to obtain. Item response options were first increased from five to seven and presented in a more intuitive order, as per Riggio et al. [27]. The original MDORS and its three subscales were found to be robustly internally consistent. The component structure also approximated the current model, with only one item loading on a different component than expected and four items being removed due to cross-loading. This confirms the value of the existing MDORS as a robust measure of human–dog relationships.

The second aim was to evaluate a version of the MDORS expanded by the addition of four items from the C/DORS created by Howell et al. [16] and modified by replacing objective response options with subjective alternatives in those items that previously had objective options. A Principal Components Analysis was conducted, following which, four items were removed. Reanalysis of the remaining 28 items produced a robust, interpretable, four-component solution, which was reproduced in a 12-item short form of the scale. Individual subscales were typically moderately positively correlated, and most were also robustly correlated with other measures of the dog–owner relationship, including validated measures from the Lexington Attachment to Pets Scale (LAPS) [4] and various single items included in this study.

Two new scales were created based on these results, named the Dog–Owner Relationship Scale 28 (DORS28) and the Dog–Owner Relationship Scale 12 (DORS12). These are available in the (Supplementary Materials File S5: DORS28 and DORS12 administration and scoring instructions). The new DORS scales are constructed similarly to the MDORS; however, the items were re-ordered to reflect the new four-component structure and facilitate the administration and scoring of the short form. Although some authors may criticize the use of the term ‘owner’ rather than ‘guardian’ or ‘carer’ in the title, it remains a commonly used term in the literature and, in our experience, captures how most people with companion or pet dogs describe their relationship. Indeed, participants in the current study were recruited on that basis; only Prolific users who had previously indicated that they owned a dog were invited to participate.

Interestingly, the comprehensive analyses conducted in this study both confirmed the original three-component nature of the relationship captured by the original MDORS and expanded upon this by reorganizing the data to enable an additional component to emerge. The four subscales included in the new measures can all be subsumed conceptually under the three original dimensions: the Perceived Costs (PCO) of living with a pet, the Perceived Emotional Closeness (PEC) that characterizes many such relationships, and the Interactions that Pet Owners typically engage in with their animals (POIs). We anticipate, however, that the new measures will provide a more nuanced understanding of human–dog relationships.

The Perceived Costs component of the MDORS was virtually unchanged regardless of how the data were analyzed and which variables or response options were included. This is a strong and reliable component not well captured in other measures of human–animal relationships. It appears mostly independent of variables like participant gender, type of residence, and dog sex, although it was significantly positively correlated with participant age, dog age, and the number of dogs currently living with the participant. Several items included in this subscale are negatively skewed, as are the overall subscales in both the 28-item and 12-item versions of the scale. Even in this sample, selected to be less dog-centric than those often obtained in this field, participants rarely agreed that their dog was difficult to look after, stopped them doing what they wanted, cost too much, or made too much mess. This speaks to the generally positive nature of human–companion dog relationships and is consistent with social exchange theory [10], which argues that only relationships where the perceived benefits outweigh the perceived costs are maintained.

Many of the items that were in the PEC subscale of the MDORS are now included in a subscale we called Emotional Reliance (DORS28-EMR and DORS12-EMR). Participants who scored highly on this subscale, as many did, appear not to be merely emotionally close to their animal but may rely on this animal to fill many basic human social needs. The extent to which this component may have become stronger quite recently, due to broader social changes associated with the aftermath of the COVID-19 pandemic, including escalating rates of social isolation, loneliness, and mental health disorders, is unknown. It is intriguing, however, that the EMR subscales are strongly negatively skewed, with means and medians close to the high end of the possible range. The participants in the current study were purposely drawn from a sample who signed up to participate in research for monetary compensation and who reported on a screening instrument that they owned a dog. They were not drawn from social media sites or dog-focused special interest groups. Nonetheless, they broadly agreed, among other similar items, that their dog helps them get through tough times and is there whenever they need to be comforted. This confirms the singular importance of companion dogs in the emotional lives of many modern adults, at least in Western cultures. Since people generally consider their pets to be a member of the family [24], it follows that they would experience a strong emotional bond, and, indeed, reliance, on them, just like they would with close human family members. However, this finding could be a reason for concern if people are relying on dogs to the exclusion of humans, as this has been associated with poorer mental health outcomes [30,31,32].

Items from the POI subscale of the MDORS split up in our analyses. Five of the items fell into a component identified as Active Engagement (DORS28-ENG and DORS12-ENG). Participants who scored highly on this subscale interact with their dogs in an active manner, taking them to visit people, in the car or on public transport, grooming them, buying them gifts, and playing games with them. In the current sample, scores on these subscales were approximately normally distributed. As expected, they were higher in participants who engaged in various dog sports and activities and tended to be lower when dogs were younger or when owners lived with multiple dogs.

The remaining items that were previously included in the POI subscale of the MDORS, plus the four items drawn from the C/DORS, fell into a fourth component that we chose to name Affectionate Engagement (DORS28-AFF and DORS12-AFF). These items reflect engagement between an owner and their dog that is less externally focused than the previous subscale and perhaps more focused on maintaining emotional closeness. Participants who scored highly on this subscale reported kissing, hugging, watching, petting, talking to, giving food treats to, and cuddling their dog. Although this factor was, as might be expected, strongly positively correlated with the Emotional Reliance factor, scores on this subscale were less skewed, suggesting that Affectionate Engagement, while common, can be statistically dissociated from Emotional Reliance, which was almost uniformly high in this sample. Consistent with this conclusion, the median score for DORS28-AFF was 5.33, whereas, for DORS28-EMR, it was 6.40 on the seven-point scale. Scores on the AFF subscales were not statistically associated with participant age or the age, weight, sex, or source of the dog, but they did differ across participant gender, with females scoring slightly higher on this subscale.

The four-component solution is intriguing. As mentioned previously, a strength of this study was the use of participants recruited from Prolific, an online provider for matching participants with researchers seeking data. This likely enabled avoidance of a dog-centric sample. It also lessened, but did not eliminate, the gender bias reported in many previous MDORS studies, where samples have commonly been overwhelmingly female [11,16]. Nonetheless, it will be important in future research to evaluate the effectiveness of the new scales in culturally diverse samples and, particularly, in samples less likely to participate in paid, online, data gathering platforms. The MDORS has previously been translated into languages other than English [13,14,27]. Whether the same four-component solution will emerge in countries other than the USA is unknown.

It will also be necessary in the future to determine whether any of the DORS subscales are associated with or predicted by specific demographic variables. Few associations were found in this study, but a comprehensive analysis of the demographic data was beyond the scope of this work and, ideally, should be conducted using an independent sample rather than the one used to develop the measures. The survey we conducted was quite long, with many items repeated twice with different response options. The presence of these items, eventually removed as redundant (i.e., all the items with objective response options), may have impacted participants’ responses, as well as preventing us from asking additional questions. It will be important in the future, for example, to determine if different training approaches affect relationship quality or if specific health or behavioral issues in dogs lead to different outcomes in terms of relationship strength.

Broadening the response options from five to seven points appeared to improve the range of scores obtained for many individual items, which tended to cluster towards the top end of the range but with more variability than a five-point scale would allow. Replacing objective response options with more subjective ones for items where this was appropriate also often, but not always, improved the psychometric properties of individual items. Given the emphasis of the MDORS, and now DORS, on measuring owner perceptions of their relationship with their companion dog, rather than the dog’s perception or any objective indicators of relationship strength, this change seems justified both statistically and conceptually. Caution should be exercised when comparing across groups, however, as it is impossible to know if the more subjective labels will be interpreted consistently. While most participants, regardless of demographic group or culture, would interpret ‘once per day’ or ‘several times per week’ in the same way, there may be more variability in how ‘extremely often’ or ‘rarely’ are understood. Regardless, it was pleasing to see that, while the previously identified problem of non-normally distributed data was not eliminated, it was reduced. Some individual items remained heavily negatively skewed, but the subscale scores were mostly within the acceptable range of ±1 for skewness and ±2 for kurtosis. The exception was the Emotional Reliance subscale, which achieved higher skewness and kurtosis scores than are desirable.

In recent years, the MDORS has been modified for use in pet species other than dogs [15,16]. In the original study, we specifically chose to create a species-specific scale, recognizing that there are features of human–dog relationships that may not be applicable to other species and that may bias some existing measures towards dog owners. It might be expected, for example, that owners of cats might be less likely to take their pets visiting or to groom them regularly, and this should not be taken as indicative that their relationship is any less rich than that of comparable dog owners. Howell et al. [16] overcame this limitation of the MDORS to some extent by identifying new items that enabled cat owners to use a version of the MDORS named the CORS. This was published in the form of a combined survey, the C/DORS, from which data could be extracted for both dog and cat owners. In this study, those additional items enabled identification of a dimension of dog ownership not previously included in the MDORS, the subscale named Affectionate Engagement. This may represent an entirely new dimension of engagement between owners and pets emerging in recent years, as emotional attachments between humans and their pets have strengthened in importance and closeness, or it may represent a dimension that once characterized cat–human relationships but is now also increasingly common in dog–human relationships. Future research will be required to accurately assess species differences and many other issues and, we would argue, should use the DORS28 when possible. While our results confirm that the MDORS is an excellent measure of human–dog relationships, able to be used with confidence when direct comparisons with existing research are required, the additional nuance provided by the DORS28 is attractive. In addition, while the DORS12 was found to be remarkably robust for a short scale, the longer scale is likely to be more sensitive to subtle differences across species or demographic groups and is recommended whenever administration of a longer survey is feasible.

## 5. Conclusions

Reassessment of the MDORS in a large sample of adult residents of the USA, selected to be less dog-centric than previous samples, both confirmed that the instrument is robust and identified various measures that could be taken to refine it. The result is two new surveys designed to assess four components of the relationships that develop between adult humans and their companion dogs: the Perceived Costs of Dog Ownership, the Emotional Reliance of the owner on the dog, the perceived frequency with which the owner demonstrates affection (Affectionate Engagement) towards their dog, and the extent of Active Engagement in shared activities by dog and owner. The component structure confirms that social exchange theory remains relevant to the field of anthrozoology but suggests that perceived benefits for dog owners mostly far outweigh any perceived costs. Further research is required to establish the applicability of the new structure to other cultures or specific demographic groups. Achieving this will be facilitated by the availability of two new measures: the Dog-Owner Relationship Scale 28 (DORS28) and the Dog-Owner Relationship Scale 12 (DORS12). These are freely available (see Supplementary Materials File S5: DORS28 and DORS12 administration and scoring instructions) and can be personalized for online use, as was done in this study.

## Figures and Tables

**Table 1:** Participant demographics (*n* = 354).

Owner Demographics		
**Age**		
Mean	40.8	
Median	38.0	
Standard deviation	13.4	
Range	17–79	
**Gender**	***n***	**%**
Female	216	61.0
Male	134	37.9
Other/prefer not to say	4	1.1
**Level of education**		
Grade 11 or 12 or below	53	15.0
Certificate/diploma	118	33.3
Bachelor’s degree	112	31.6
Master’s/PhD	68	19.2
Other/Prefer not to say	3	0.8
**Home location**		
Suburban	165	46.6
Regional city	27	7.6
Rural	49	13.8
Urban	73	20.6
Country town	40	11.3
**Dwelling type**		
House	269	76.0
Unit/apartment	61	17.2
Semi-detached	21	5.3
Trailer/RV	3	0.8
**Amount of outside space in dwelling**		
Large	38	10.7
Medium	175	49.4
Small	128	36.2
None	13	3.7
**Employment type**		
Full-time paid work	193	54.5
Unemployed	28	7.9
Home duties	21	5.9
Unable to work	13	3.7
Part-time paid work	58	16.4
Retired	17	4.8
Student	15	4.2
Other/Prefer not to say	9	2.5
**Number of adults in home**		
1	71	20.1
2	162	45.8
3	76	21.5
4 or more	45	12.7
**Number of children in home**		
0	210	59.3
1	72	20.3
2	47	13.3
3	22	6.2
4 or more	3	0.8
**Marital status**		
Separated/divorced/widowed	41	11.6
Married	153	43.2
Single/never married	118	33.3
De facto/Living together	36	10.2
Prefer not to say	6	1.7

**Table 2:** Dog demographics, activities that participants engaged in with their dogs, and participant satisfaction with their dog’s behavior and health (*n* = 354).

Dog Demographics	*n*	%
**Current dog age**		
Less than 1 year	18	5.1
1–2 years	67	18.9
3–8 years	177	50.0
9–12 years	61	17.2
13+ years	30	8.5
Don’t know	1	0.3
**Dog age at acquisition**		
Less than 1 month	34	9.6
1–4 months	207	58.5
5–12 months	52	14.7
1–3 years	38	10.7
Over 3 years	20	5.6
Don’t know	3	0.8
**Sex of dog**		
Male Entire	62	17.5
Male Desexed	130	36.7
Female Entire	40	11.3
Female Desexed	120	33.9
Don’t Know	2	0.6
**Dog source**		
Pet Shop	14	4.0
Breeder	99	28.0
Shelter/Rescue	95	26.8
Friend/Family	101	28.5
Other	9	2.5
Found	15	4.2
Bred Myself	10	2.8
Gift	6	1.7
Inherited	5	1.4
**Dog spends time**		
Inside the house	251	70.9
Both inside and outside equally	91	25.7
Outside the house	12	3.4
**Activities with dog**		
Dog sports	84	23.7
Tracking	24	6.8
Obedience training	109	30.8
Search and Rescue	14	4.0
Animal Assisted Intervention	8	2.3
Tricks	121	34.2
General	328	92.7
Other	19	5.4
None	8	2.3
**Satisfaction with dog’s behavior**		
Mean	4.26	
Median	4	
Standard deviation	0.86	
Range	1–5	
**Satisfaction with dog’s health**		
Mean	4.26	
Median	5	
Standard deviation	0.95	
Range	1–5	
**Relationship quality relative to other owners**		
Mean	5.83	
Median	6	
Standard deviation	1.27	
Range	2–7	

**Table 3:** The 49 E-DORS items, including source, descriptive statistics, and whether they were retained in the DORS28 or DORS12.

MDORS Item	C/DORS Item	E-DORS Item	Reverse-Scored	Response Option Text When Item Was Repeated	Range	Mean	SD	Median	Skewness	Kurtosis	Retained in DORS28	Retained in DORS12
1 *	1	1	Yes		1–7	5.69	1.37	6.00	−0.98	0.21	Yes	
2	2	3			1–7	5.87	1.33	6.00	−1.43	2.04		
3	3	4	Yes		1–7	5.45	1.77	6.00	−1.04	−0.13	Yes	
4	4	5		Objective	1–7	4.24	1.59	5.00	−0.61	−0.13		
4	4	11		Subjective	1–7	4.62	1.72	5.00	−0.45	−0.69	Yes	Yes
5	5	7			1–7	5.25	1.75	6.00	−0.90	−0.17		
6	6	9	Yes		1–7	5.54	1.58	6.00	−1.03	0.003	Yes	
7	7	10		Objective	1–7	4.14	1.32	4.00	−0.49	0.18		
7	7	2		Subjective	1–7	5.00	1.42	5.00	−0.51	−0.47	Yes	
8*	8	12	Yes		1–7	5.94	1.37	6.00	−1.43	1.35	Yes	Yes
9	27	42		Objective	1–7	3.05	1.51	3.00	0.28	−0.71		
9	27	21		Subjective	1–7	3.43	1.60	3.00	0.41	−0.52	Yes	Yes
10	10	15	Yes		1–7	5.55	1.62	6.00	−0.93	−0.32	Yes	Yes
11	11	17	Yes		1–7	5.71	1.54	6.00	−1.11	0.25	Yes	
12 *	12/31	18		Objective	1–7	2.96	1.07	3.00	0.45	−0.08		
12 *	12/31	37		Subjective	1–7	3.91	1.31	4.00	0.39	−0.21	Yes	
13	33	49			1–7	5.98	1.21	6.00	−1.6	3.17	Yes	Yes
14	28	43		Objective	1–7	4.22	1.19	5.00	−0.64	0.57		
14	28	22		Subjective	1–7	5.17	1.38	5.00	−0.43	−0.57	Yes	
15	13	20		Objective	1–7	3.67	2.02	4.00	0.05	−1.21		
15	13	6		Subjective	1–7	3.60	1.89	3.00	0.19	−1.08	Yes	
16	14	23	Yes	Objective	1–7	6.16	1.40	7.00	−1.7	2.18		
16	14	33	Yes	Subjective	1–7	5.95	1.39	6.00	−1.6	2.53	Yes	
17 *	29	44		Objective	1–7	3.41	1.59	3.00	0.09	−0.93		
17 *	29	34		Subjective	1–7	3.79	1.63	3.00	0.24	−0.76	Yes	Yes
18	16	26	Yes	Objective	1–7	6.49	1.04	7.00	−2.24	4.59		
18	16	45	Yes	Subjective	1–7	5.95	1.19	6.00	−1.48	2.57	Yes	Yes
19	17	27			1–7	5.41	1.63	6.00	−0.98	0.27		
20	32	48		Objective	1–7	3.83	1.37	4.00	0.09	−0.52		
20	32	40		Subjective	1–7	4.54	1.30	5.00	−0.24	−0.48	Yes	Yes
21	18	29			1–7	6.27	1.19	7.00	−2.04	4.4	Yes	
22	19	30	Yes	Objective	1–7	6.63	1.09	7.00	−3.12	9.22		
22	19	16	Yes	Subjective	2–7	6.36	1.15	7.00	−2.41	6.3	Yes	
23	20	31			1–7	5.88	1.40	6.00	−1.52	2.22	Yes	
24	30	46		Objective	1–7	4.62	1.45	5.00	−0.74	0.61		
24	30	25		Subjective	1–7	5.33	1.49	6.00	−0.90	0.36	Yes	Yes
25	22	35			1–7	6.13	1.11	6.00	−1.7	3.78	Yes	Yes
26	23	36		Objective	1–7	5.34	1.44	5.00	−0.77	0.57		
26	23	47		Subjective	1–7	5.75	1.31	6.00	−1.22	1.43		
27	24	38			1–7	6.02	1.21	6.00	−1.52	2.57	Yes	Yes
28	25	39			1–7	5.68	1.38	6.00	−1.15	0.96	Yes	
-	9	13		Objective	1–7	5.08	1.31	5.00	−0.48	0.70		
-	9	28		Subjective	1–7	5.67	1.26	6.00	−1.01	0.95	Yes	
-	15	24		Objective	1–7	5.24	1.38	5.00	−0.84	0.95		
-	15	14		Subjective	1–7	5.66	1.30	6.00	−0.87	0.15	Yes	
-	21	32		Objective	1–7	4.75	1.42	5.00	−0.68	0.69		
-	21	8		Subjective	1–7	5.45	1.44	6.00	−0.98	0.59	Yes	Yes
-	26	41		Objective	1–7	5.50	1.10	5.00	−0.59	1.13		
-	26	19		Subjective	2–7	6.20	1.05	6.00	−1.58	2.37	Yes	

* Question text altered slightly from MDORS to improve conceptual interpretability or readability.

**Table 4:** Principal Components Analysis results showing items retained in each of four components, their source, and component loadings, with the strongest loading bolded, for each item in both 28-item and 12-items versions of the Dog Owner Relationship Scale. All items used subjective response options.

Item	Component Loadings on DORS-28				Component Loadings on DORS-12			
	AFF	PCO	EMR	ENG	AFF	PCO	EMR	ENG
How often do you hug [petname]? (MDORS24)	**0.850**	0.005	0.044	0.049	**0.865**	−0.003	0.013	0.010
How often do you kiss [petname]? (MDORS4)	**0.784**	0.013	0.114	0.072	**0.805**	0.031	−0.073	0.015
How often do you cuddle [petname]? (CDORS21)	**0.768**	−0.056	−0.109	0.008	**0.738**	−0.036	0.159	−0.012
How often do you pet [petname]? (CDORS26)	**0.740**	0.048	−0.061	−0.157				
How often do you talk to [petname]? (CDORS15)	**0.564**	−0.021	−0.113	−0.008				
How often do you spend time enjoying watching [petname]? (CDORS9)	**0.557**	0.195	−0.175	0.089				
How traumatic do you think it will be for you when [Petname] dies? (MDORS28)	**0.557**	0.149	−0.192	−0.012				
How often do you give [petname] food treats? (MDORS14)	**0.378**	−0.030	0.044	0.170				
How often do you tell [petname] things you do not tell anyone else? (MDORS15)	**0.328**	−0.011	−0.214	0.148				
It bothers me that [Petname] stops me doing things I enjoyed before I owned him/her. (MDORS8)	0.005	**0.821**	0.103	0.067	0.055	**0.866**	−0.073	0.078
How often does [Petname] stop you doing things you want to? (MDORS18)	−0.070	**0.815**	0.040	−0.112	−0.011	**0.817**	0.020	0.024
It is annoying that I sometimes have to change my plans because of [Petname]. (MDORS10)	−0.057	**0.801**	0.034	0.040	−0.039	**0.737**	0.056	−0.115
How often do you feel that looking after [Petname] is a chore? (MDORS16)	0.036	**0.770**	−0.052	−0.112				
How often do you feel that having [Petname] is more trouble than it is worth? (MDORS22)	0.131	**0.652**	−0.145	−0.091				
There are major aspects of owning [Petname] that I do not like. (MDORS3)	0.046	**0.634**	−0.165	0.050				
[Petname] costs too much money. (MDORS11)	0.074	**0.557**	0.048	−0.011				
How difficult is it to look after [Petname]? (MDORS1)	−0.044	**0.531**	−0.006	0.023				
[Petname] makes too much mess. (MDORS6)	−0.012	**0.503**	−0.074	0.122				
[Petname] provides me with constant companionship. (MDORS25)	0.029	0.032	**−0.854**	0.003	−0.026	0.020	**0.940**	0.004
[Petname] is there whenever I need to be comforted. (MDORS27)	0.075	0.015	**−0.796**	−0.006	0.112	0.031	**0.735**	−0.006
[Petname] is constantly attentive to me. (MDORS13)	−0.042	−0.041	**−0.744**	0.053	0.004	−0.018	**0.725**	0.010
If everyone else left me, [petname] would still be there for me. (MDORS21)	0.017	0.094	**−0.678**	0.014				
[Petname] helps me get through tough times. (MDORS23)	0.272	0.087	**−0.558**	0.042				
How often do you take [petname] in the car, on your bike, or on public transport? (MDORS17)	0.035	0.000	0.012	**0.790**	0.113	−0.012	−0.023	**0.720**
How often do you take [petname] to visit people? (MDORS9)	−0.033	−0.015	−0.010	**0.782**	−0.061	−0.026	−0.014	**0.886**
How often do you groom [petname]? (MDORS20)	0.016	0.065	−0.273	**0.426**	0.039	0.082	0.280	**0.375**
How often do you play games with [petname]? (MDORS7)	0.197	0.026	−0.133	**0.364**				
How often do you buy [petname] gifts? (MDORS12	0.267	0.034	−0.011	**0.348**				
[Petname] gives me a reason to get up in the morning. (MDORS2)	Removed due to cross-loading							
I wish [petname] and I never had to be apart (MDORS5)	Removed due to cross-loading							
I would like to have [petname] near me all the time. (MDORS19)	Removed due to cross-loading							
How often do you have [Petname] with you while relaxing, i.e., watching TV? (MDORS26)	Removed due to cross-loading							

Legend: PCO—Perceived Costs; AFF—Affectionate Engagement; EMR—Emotional Reliance; ENG—Active Engagement. Abbreviated item names indicate the source of the item (MDORS vs. CDORS and original item number).

**Table 5:** Properties for the measures derived from the DORS28 and DORS12.

	DORS28					DORS12				
	PCO	AFF	EMR	ENG	TOT	PCO	AFF	EMR	ENG	TOT
% variance explained	13.39	35.11	5.35	5.12	58.98	18.43	9.92	38.54	9.01	75.90
Cronbach’s α	0.887	0.886	0.892	0.781	0.923	0.839	0.856	0.855	0.742	0.838
Mean	5.79	5.26	6.06	4.13	21.2	5.81	5.13	6.04	3.92	20.9
Standard Deviation	1.05	1.05	1.03	1.07	3.23	1.22	1.37	1.04	1.23	3.41
Median	6.11	5.33	6.40	4.20	21.7	6.33	5.33	6.33	4.00	21.3
Possible Range	1–7	1–7	1–7	1–7	4–28	1–7	1–7	1–7	1–7	4–28
Min. Score Obtained	2.00	1.78	1.00	1.40	7.24	1.33	1.00	1.00	1.00	8.00
Max. Score Obtained	7.00	7.00	7.00	7.00	28.00	7.00	7.00	7.00	7.00	28.00
Skewness	−1.02	−0.71	−1.52	−0.07	−0.79	−1.09	−0.765	−1.52	0.013	−0.610
Kurtosis	0.67	0.48	3.07	−0.36	1.08	0.528	0.280	2.95	−0.502	0.304

Legend: PCO—Perceived Costs; AFF—Affectionate Engagement; EMR—Emotional Reliance; ENG—Active Engagement; TOT—total score.

**Table 6:** Spearman’s *r* values correlating DORS28 subscales with other variables (all *df* = 350–352). Cell shading indicates a large (>0.5) effect size.

Variable	DORS28-PCO	DORS28-AFF	DORS28-EMR	DORS28-ENG	DORS28-TOT
DORS28-PCO	-	0.371 ***	0.494 ***	0.212 ***	0.670 ***
DORS28-AFF		-	0.644 ***	0.529 ***	0.810 ***
DORS28-EMR			-	0.441 ***	0.822 ***
DORS28-ENG				-	0.713 ***
DORS12-PCO	0.883 ***	0.308 ***	0.410 ***	0.127 *	0.563 ***
DORS12-AFF	0.301 ***	0.909 ***	0.529 ***	0.493 ***	0.709 ***
DORS12-EMR	0.447 ***	0.601 ***	0.956 ***	0.401 ***	0.762 ***
DORS12-ENG	0.165 **	0.455 ***	0.391 ***	0.934 ***	0.638 ***
DORS12-TOT	0.623 ***	0.794 ***	0.760 ***	0.704 ***	0.952 ***
LAPS-1	0.475 ***	0.606 ***	0.760 ***	0.457 ***	0.740 ***
LAPS-2	0.424 ***	0.613 ***	0.731 ***	0.445 ***	0.716 ***
LAPS-3	0.450 ***	0.601 ***	0.686 ***	0.356 ***	0.680 ***
LAPS-TOTAL	0.493 ***	0.662 ***	0.800 ***	0.465 ***	0.782 ***
Dog Behavior Satisfaction	0.481 ***	0.242 ***	0.313 ***	0.261 ***	0.413 ***
Dog health Satisfaction	0.219 ***	0.110 *	0.176 ***	0.192 ***	0.219 **
Relative to other owners	0.385 ***	0.480 ***	0.530 ***	0.431 ***	0.586 ***
Dog Bond	0.352 ***	0.535 ***	0.664 ***	0.388 ***	0.614 ***
Participant age	0.221 ***	0.026	0.056	−0.003	0.091
Level of education	−0.008	0.089	0.023	0.231 ***	0.119 *
# of adults in home	−0.089	−0.055	−0.174 **	−0.057	−0.136 *
# of children in home	−0.126 *	−0.108 *	−0.160 **	−0.015	−0.147 **
# of dogs in home	0.149 **	0.018	0.014	−0.121 *	0.010
Current age of dog	0.126 *	0.019	0.006	−0.217 ***	−0.019
Dog age at acquisition	0.027	0.058	0.055	−0.092	0.021
Dog weight	−0.052	−0.011	0.079	0.007	0.011

Note. * *p* < 0.05, ** *p* < 0.01, and *** *p* < 0.001; DORS—Dog Owner Relationship Scale; PCO—Perceived Costs; AFF—Affectionate Engagement; EMR—Emotional Reliance; ENG—Active Engagement; TOT—total score; LAPS—Lexington Attachment to Pets Scale.

## Data Availability

The data collected for this study are available upon request from the corresponding author, provided that approval to release the data is obtained from the La Trobe University Human Ethics Committee.

## Supplementary Materials

The following supporting information can be downloaded at https://www.mdpi.com/article/10.3390/ani15050632/s1: File S1: Survey. File S2: Analysis of the original 28-item MDORS. File S3: Analysis of the expanded and modified 32-item E-DORS. File S4: DORS28 and DORS12 subscale properties and relationships with other variables. File S5: DORS28 and DORS12 administration and scoring instructions.
