# Supplementary material for: New 28-Item and 12-Item Dog Owner Relationship Scales: Contemporary Versions of the MDORS with a Revised Four-Component Structure
Source: Animals (Basel). 2025 Feb 21;15(5):632. doi: 10.3390/ani15050632 (PMC11898123; doi:10.3390/ani15050632)
Supplement: Supplementary file 1 [file animals-15-00632-s001.zip › File S2 - Analysis of the original 28-item MDORS.pdf]

File S2: Analysis of the original 28-item MDORS

Analysis A: Internal Consistency Analysis - 28-item MDORS with original objective response options

Scale	Mean	SD	Cronbach's α	McDonald's ω
28-item MDORS	5.18	0.784	0.913	0.918
9-item PO subscale	5.91	0.991	0.861	0.868
9-item POI subscale	3.98	0.910	0.827	0.830
10-item PEC subscale	5.62	1.07	0.909	0.921

Analysis B: PCA - 28-item MDORS with original objective response options, number of components selected using Kaiser's criterion

Total Variance Explained

	Initial Eigenvalues			Extraction Sums of Squared Loadings			Rotation Sums of Squared Loadings[1]
Factor	Total	% of variance	Cumulative %	Total	% of variance	Cumulative %	Total
1	9.103	32.512	32.512	8.640	30.857	30.857	6.504
2	3.870	13.821	46.333	3.407	12.167	43.024	4.911
3	1.714	6.121	52.454	1.304	4.659	47.683	3.777
4	1.187	4.241	56.695	0.721	2.577	50.259	4.930
5	1.049	3.746	60.441	0.748	2.671	52.930	5.141
6	1.013	3.617	64.057	0.597	2.131	55.061	2.617
7	0.854	3.050	67.107				
8	0.818	2.921	70.028				
9	0.751	2.682	72.710				
10	0.697	2.488	75.198				
11	0.622	2.222	77.420				
12	0.604	2.156	79.576				
13	0.578	2.063	81.639				
14	0.525	1.874	83.513				
15	0.495	1.769	85.283				
16	0.476	1.700	86.982				
17	0.448	1.599	88.582				
18	0.415	1.484	90.065				
19	0.410	1.466	91.531				
20	0.361	1.289	92.820				
21	0.346	1.235	94.056				
22	0.314	1.120	95.176				
23	0.298	1.066	96.241				
24	0.266	0.952	97.193				
25	0.224	0.801	97.994				

26	0.203	0.725	98.719				
27	0.187	0.667	99.386				
28	0.172	0.614	100				

1. When components are correlated, sums of squared loadings cannot be added to obtain a total variance

Scree Plot

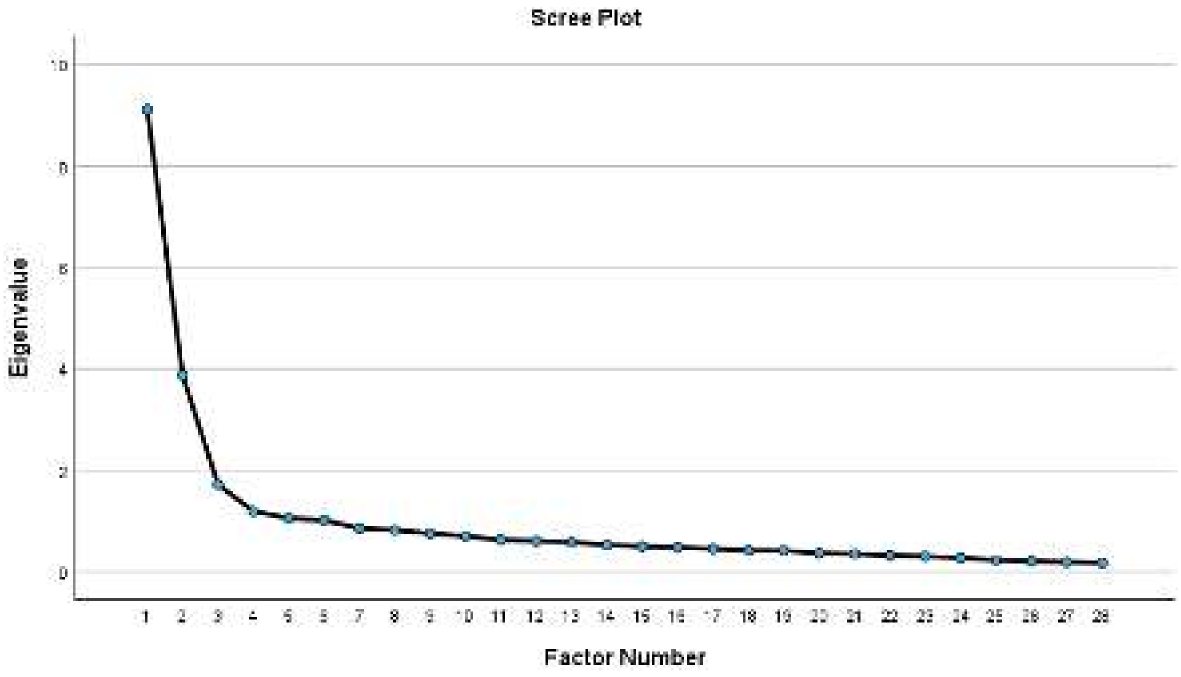

Parallel Analysis

Number of variables: 28, Number of subjects: 354, Number of replications: 100

Eigenvalue #	Initial Eigenvalue	Random Eigenvalue	Standard Deviation
1	**9.103**	1.5622	.0424
2	**3.870**	1.4800	.0352
3	**1.714**	1.4190	.0258
4	1.187	1.3648	.0243
5	1.049	1.3147	.0257
6	1.013	1.2709	.0216
7	0.854	1.2306	.0212
8	0.818	1.1914	. 0251
9	0.751	1.1545	.0219

Notes: truncated at 9 rows, bold type denotes where initial eigenvalue exceeds random eigenvalue. Generated using Monte Carlo PCA for Parallel Analysis, Version 3.0, ©2000-2020 by Marley W. Watkins. All rights reserved.

Analysis C: PCA - 28-item MDORS with original objective response options, number of factors informed by scree plot and parallel analysis, fixed at 3.

Pattern Matrix

Item Source	1	2	3
MDORS25	**0.887**	0.005	-0.076
MDORS27	**0.858**	0.029	-0.021
MDORS23	**0.745**	-0.084	0.111
MDORS13	**0.732**	0.080	-0.039
MDORS21	**0.729**	-0.030	-0.030
MDORS19	**0.558**	-0.166	0.235
MDORS5	**0.547**	-0.124	0.181
MDORS2	**0.499**	-0.199	0.244
MDORS28	**0.498**	-0.171	0.244
MDORS26_O	**0.450**	0.044	0.222
MDORS15_O	**0.347**	0.034	**0.332**
MDORS8_RS	-0.097	**-0.830**	0.053
MDORS10_RS	-0.101	**-0.828**	0.041
MDORS3_RS	0.206	**-0.606**	0.072
MDORS16_RS_O	0.196	**-0.603**	**-0.306**
MDORS18_RS_O	0.205	**-0.536**	**-0.450**
MDORS11_RS	0.001	**-0.533**	0.031
MDORS1_RS	-0.068	**-0.529**	0.079
MDORS22_RS_O	0.251	**-0.528**	**-0.305**
MDORS6_RS	0.040	**-0.528**	0.142
MDORS9_O	-0.085	-0.031	**0.688**
MDORS17_O	0.045	0.031	**0.655**
MDORS12_O	0.027	-0.075	**0.573**
MDORS7_O	0.107	0.060	**0.536**
MDORS24_O	0.261	-0.095	**0.521**
MDORS14_O	0.048	-0.018	**0.476**
MDORS4_O	0.199	-0.122	**0.476**
MDORS20_O	0.149	-0.039	**0.421**

Note. Maximum Likelihood Extraction method was used in combination with a Direct Oblimin rotation with Kaiser normalization; item names indicate original source, whether item was reverse scored and response-option type; bold text conveys loadings exceeding ± 0.3; rotation converged in 9 iterations.

Analysis D: PCA - 28-item MDORS with original objective response options, number of factors fixed at 3, four cross loaded items removed.

Pattern Matrix

Item Source	1	2	3
MDORS25	**0.894**	-0.005	-0.088
MDORS27	**0.872**	0.012	-0.049
MDORS13	**0.745**	0.046	-0.075
MDORS21	**0.741**	-0.041	-0.050
MDORS23	**0.738**	-0.061	0.119
MDORS19	**0.568**	-0.173	0.179
MDORS5	**0.555**	-0.119	0.142
MDORS2	**0.487**	-0.162	0.251
MDORS28	**0.476**	-0.117	0.274
MDORS26_O	**0.424**	0.076	0.260
MDORS10_RS	-0.070	**-0.883**	-0.073
MDORS8_RS	-0.062	**-0.833**	-0.029
MDORS3_RS	0.247	**-0.593**	-0.002
MDORS1_RS	-0.032	**-0.524**	0.010
MDORS11_RS	0.034	**-0.522**	-0.023
MDORS6_RS	0.065	**-0.505**	0.095
MDORS17_O	-0.036	0.077	**0.731**
MDORS9_O	-0.151	0.002	**0.729**
MDORS24_O	0.201	-0.038	**0.589**
MDORS12_O	-0.019	-0.058	**0.576**
MDORS7_O	0.046	0.079	**0.575**
MDORS4_O	0.148	-0.072	**0.526**
MDORS14_O	0.021	-0.010	**0.463**
MDORS20_O	0.120	-0.060	**0.395**

Note. Maximum Likelihood Extraction method was used in combination with a Direct Oblimin rotation with Kaiser normalization; item names indicate original source, whether item was reverse scored and response-option type; bold text conveys loadings exceeding ± 0.3; rotation converged in 6 iterations.

Structure Matrix

Item Source	1	2	3
MDORS25	0.850	-0.409	0.378
MDORS27	0.841	-0.390	0.403
MDORS23	0.829	-0.434	0.516
MDORS19	0.743	-0.478	0.510
MDORS21	0.734	-0.381	0.344
MDORS2	0.694	-0.443	0.538
MDORS5	0.685	-0.410	0.456
MDORS13	0.684	-0.291	0.304
MDORS28	0.674	-0.398	0.546
MDORS26_O	0.523	-0.178	0.465
MDORS10_RS	0.309	-0.835	0.073
MDORS8_RS	0.317	-0.798	0.111

MDORS3_RS	0.526	-0.709	0.248
MDORS6_RS	0.353	-0.555	0.233
MDORS11_RS	0.268	-0.533	0.102
MDORS1_RS	0.220	-0.510	0.101
MDORS24_O	0.526	-0.254	0.701
MDORS17_O	0.308	-0.057	0.696
MDORS9_O	0.227	-0.076	0.650
MDORS4_O	0.456	-0.250	0.618
MDORS7_O	0.308	-0.061	0.583
MDORS12_O	0.308	-0.168	0.578
MDORS14_O	0.266	-0.115	0.475
MDORS20_O	0.354	-0.198	0.470

Note. Maximum Likelihood Extraction method was used in combination with a Direct Oblimin rotation with Kaiser normalization; item names indicate original source, whether item was reverse scored and response-option type.

KMO and Bartlett's Test

Kaiser-Meyer-Olkin Measure of Sampling Adequacy		0.912
Bartlett's Test of Sphericity	Approx. Chi-Squar	4310.798
	df	276
	Sig.	<0.001

Total Variance Explained

	Initial Eigenvalues			Extraction Sums of Squared Loadings			Rotation Sums of Squared Loadings[1]
Factor	Total	% of variance	Cumulative %	Total	% of variance	Cumulative %	Total
1	8.495	35.397	35.397	7.963	33.181	33.181	7.090
2	2.755	11.480	46.876	2.181	9.087	42.268	4.431
3	1.703	7.095	53.971	1.283	5.346	47.613	5.110
4	1.144	4.765	58.736				
5	1.015	4.228	62.965				
6	0.910	3.790	66.755				
7	0.778	3.242	69.997				
8	0.751	3.129	73.126				
9	0.693	2.889	76.015				
10	0.607	2.528	78.543				
11	0.586	2.443	80.986				
12	0.529	2.202	83.189				
13	0.507	2.112	85.300				
14	0.473	1.972	87.272				
15	0.433	1.803	89.075				
16	0.410	1.709	90.784				

17	0.397	1.656	92.440				
18	0.352	1.465	93.905				
19	0.323	1.346	95.251				
20	0.284	1.183	96.434				
21	0.268	1.117	97.551				
22	0.212	0.884	98.435				
23	0.195	0.811	99.246				
24	0.181	0.754	100.00				

When components are correlated, sums of squared loadings cannot be added to obtain a total variance

Component Correlation Matrix

Factor	1	2	3
1	1.000	-.472	.521
2		1.000	-.206
