# Supplementary material for: New 28-Item and 12-Item Dog Owner Relationship Scales: Contemporary Versions of the MDORS with a Revised Four-Component Structure
Source: Animals (Basel). 2025 Feb 21;15(5):632. doi: 10.3390/ani15050632 (PMC11898123; doi:10.3390/ani15050632)
Supplement: Supplementary file 1 [file animals-15-00632-s001.zip › File S3 - Analysis of the expanded and modified 32-item E-DORS.pdf]

File S3: Analysis of the expanded and modified 32-item DORS

Analysis A: PCA - 32-item EDORS with subjective response options, number of factors selected using Kaiser's criterion

Total Variance Explained

	Initial Eigenvalues			Extraction Sums of Squared Loadings			Rotation Sums of Squared Loadings[1]
Factor	Total	% of variance	Cumulative %	Total	% of variance	Cumulative %	Total
1	11.784	36.825	36.825	11.335	35.421	35.421	7.875
2	3.834	11.981	48.806	3.377	10.554	45.975	6.422
3	1.552	4.851	53.657	1.092	3.412	49.387	8.035
4	1.442	4.506	58.163	0.886	2.768	52.155	5.337
5	1.197	3.740	61.903	0.938	2.931	55.086	4.815
6	0.967	3.023	64.926				
7	0.872	2.724	67.650				
8	0.828	2.587	70.237				
9	0.761	2.378	72.615				
10	0.689	2.154	74.770				
11	0.636	1.988	76.757				
12	0.603	1.884	78.641				
13	0.576	1.800	80.441				
14	0.570	1.782	82.223				
15	0.496	1.550	83.773				
16	0.472	1.474	85.247				
17	0.461	1.441	86.688				
18	0.435	1.359	88.047				
19	0.401	1.254	89.301				
20	0.387	1.208	90.509				
21	0.359	1.123	91.632				
22	0.341	1.065	92.697				
23	0.315	0.985	93.682				
24	0.293	0.914	94.597				
25	0.282	0.882	95.478				
26	0.262	0.819	96.298				
27	0.240	0.750	97.047				
28	0.220	0.687	97.734				
29	0.200	0.626	98.360				
30	0.190	0.593	98.953				
31	0.176	0.551	99.504				
32	0.159	0.496	######				

1. When components are correlated, sums of squared loadings cannot be added to obtain a total variance

Scree Plot

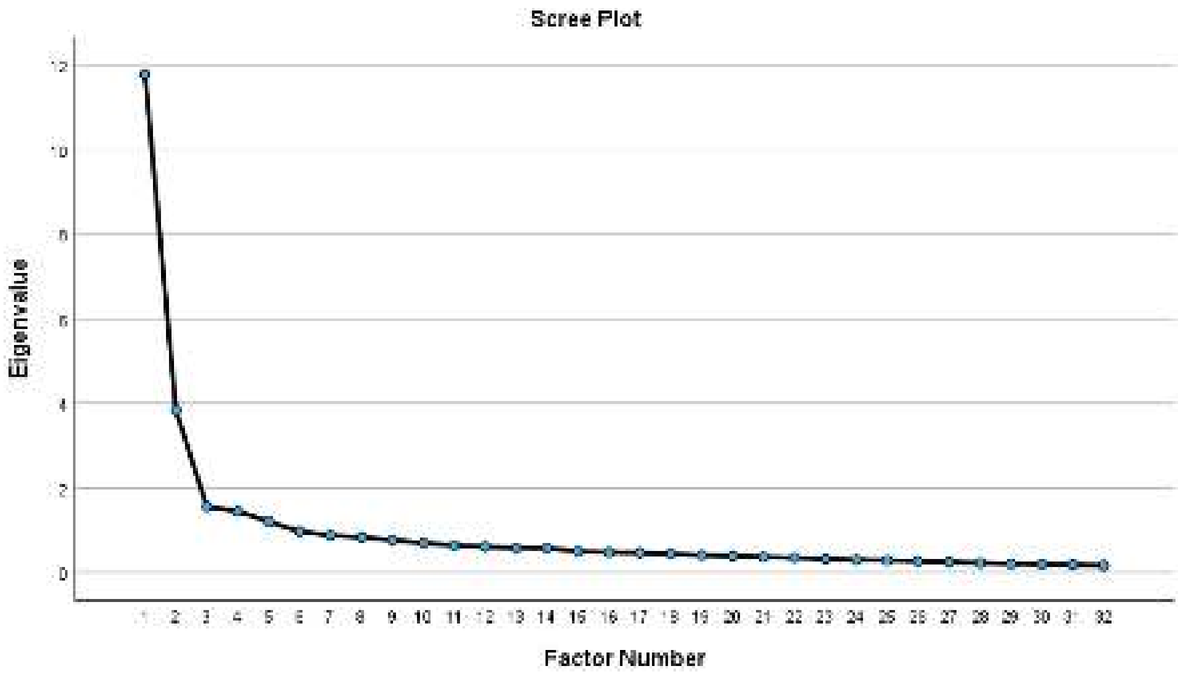

Parallel Analysis

Number of variables: 32, Number of subjects: 354, Number of replications: 100

Eigenvalue #	Initial Eigenvalue	Random Eigenvalue	Standard Deviation
1	**11.784**	1.6003	0.0398
2	**3.834**	1.5282	0.0328
3	**1.552**	1.4657	0.0319
4	**1.442**	1.4159	0.0276
5	1.197	1.3683	0.0249

Notes: truncated at 5 rows, bold type denotes where initial eigenvalue exceeds random eigenvalue. Generated using Monte Carlo PCA for Parallel Analysis, Version 3.0, ©2000-2020 by Marley W. Watkins. All rights reserved.

Analysis B: PCA - 32-item EDORS with subjective response options, number of factors informed by scree plot and parallel analysis, fixed at 4.

Pattern Matrix

Item Source	1	2	3	
MDORS24_S	**0.837**	0.013	0.052	0.065
MDORS4_S	**0.778**	0.021	0.121	0.090
CDORS21_S	**0.765**	-0.050	-0.106	0.018

CDORS26_S	**0.735**	0.055	-0.048	-0.152
CDORS15_S	**0.564**	-0.021	-0.110	-0.005
CDORS9_S	**0.547**	0.202	-0.174	0.104
MDORS28	**0.544**	0.162	-0.196	0.005
MDORS14_S	**0.378**	-0.031	0.054	0.174
MDORS15_S	**0.317**	-0.003	-0.219	0.165
MDORS8_RS	0.003	**0.825**	0.109	0.072
MDORS18_RS_S	-0.066	**0.810**	0.042	-0.119
MDORS10_RS	-0.059	**0.803**	0.040	0.043
MDORS16_RS_S	0.034	**0.772**	-0.056	-0.114
MDORS22_RS_S	0.126	**0.658**	-0.149	-0.090
MDORS3_RS	0.039	**0.636**	-0.170	0.054
MDORS11_RS	0.073	**0.554**	0.046	-0.014
MDORS1_RS	-0.043	**0.526**	-0.005	0.017
MDORS6_RS	-0.014	**0.495**	-0.082	0.116
MDORS25	0.013	0.049	**-0.841**	0.012
MDORS27	0.051	0.026	**-0.801**	0.002
MDORS13	-0.058	-0.032	**-0.742**	0.056
MDORS21	-0.006	0.104	**-0.689**	0.020
MDORS23	0.248	0.100	**-0.573**	0.057
MDORS26_S	**0.398**	-0.136	**-0.402**	0.021
MDORS19	**0.314**	0.180	**-0.355**	0.139
MDORS2	**0.307**	0.186	**-0.338**	0.095
MDORS5	**0.307**	0.158	**-0.336**	0.082
MDORS17_S	0.019	-0.011	0.020	**0.800**
MDORS9_S	-0.049	-0.026	-0.001	**0.791**
MDORS20_S	0.002	0.064	-0.269	**0.434**
MDORS7_S	0.182	0.025	-0.118	**0.368**
MDORS12_S	0.257	0.034	-0.003	**0.362**

Note. Maximum Likelihood Extraction method was used in combination with a Direct Oblimin rotation with Kaiser normalization; item names indicate original source, whether item was reverse scored and response-option type; bold text conveys loadings exceeding ± 0.3; rotation converged in 8 iterations.

Analysis C: PCA - 32-item EDORS with subjective response options, number of factors fixed at 4, four cross loaded items removed.

Parallel Analysis

Number of variables: 28, Number of subjects: 354, Number of replications: 100

Eigenvalue #	Initial Eigenvalue	Random Eigenvalue	Standard Deviation
1	**9.832**	1.5554	0.0422
2	**3.751**	1.4729	0.0374
3	**1.497**	1.4139	0.0290
4	**1.434**	1.3616	0.0278
5	0.966	1.3636	0.0249

Notes: truncated at 5 rows, bold type denotes where initial eigenvalue exceeds random eigenvalue. Generated using Monte Carlo PCA for Parallel Analysis, Version 3.0, ©2000-2020 by Marley W. Watkins. All rights reserved.

Pattern Matrix

Item Source	1 (AFF)	2 (PCO)	3 (EMR)	4 (ENG)
MDORS24_S	**0.850**	0.005	0.044	0.049
MDORS4_S	**0.784**	0.013	0.114	0.072
CDORS21_S	**0.768**	-0.056	-0.109	0.008
CDORS26_S	**0.740**	0.048	-0.061	-0.157
CDORS15_S	**0.564**	-0.021	-0.113	-0.008
CDORS9_S	**0.557**	0.195	-0.175	0.089
MDORS28	**0.557**	0.149	-0.192	-0.012
MDORS14_S	**0.378**	-0.030	0.044	0.170
MDORS15_S	**0.328**	-0.011	-0.214	0.148
MDORS8_RS	0.005	**0.821**	0.103	0.067
MDORS18_RS_S	-0.070	**0.815**	0.040	-0.112
MDORS10_RS	-0.057	**0.801**	0.034	0.040
MDORS16_RS_S	0.036	**0.770**	-0.052	-0.112
MDORS22_RS_S	0.131	**0.652**	-0.145	-0.091
MDORS3_RS	0.046	**0.634**	-0.165	0.050
MDORS11_RS	0.074	**0.557**	0.048	-0.011
MDORS1_RS	-0.044	**0.531**	-0.006	0.023
MDORS6_RS	-0.012	**0.503**	-0.074	0.122
MDORS25	0.029	0.032	**-0.854**	0.003
MDORS27	0.075	0.015	**-0.796**	-0.006
MDORS13	-0.042	-0.041	**-0.744**	0.053
MDORS21	0.017	0.094	**-0.678**	0.014
MDORS23	0.272	0.087	**-0.558**	0.042
MDORS17_S	0.035	0.000	0.012	**0.790**
MDORS9_S	-0.033	-0.015	-0.010	**0.782**
MDORS20_S	0.016	0.065	-0.273	**0.426**
MDORS7_S	0.197	0.026	-0.133	**0.364**
MDORS12_S	0.267	0.034	-0.011	**0.348**

Note. Maximum Likelihood Extraction method was used in combination with a Direct Oblimin rotation with Kaiser normalization; item names indicate original source, whether item was reverse scored and response-option type; bold text conveys loadings exceeding ± 0.3; rotation converged in 7 iterations.

Structure Matrix

Item Source	1	2	3	
MDORS24_S	0.850	0.245	-0.489	0.459
CDORS21_S	0.821	0.224	-0.550	0.430
CDORS9_S	0.766	0.448	-0.632	0.451
MDORS4_S	0.755	0.204	-0.391	0.423
CDORS26_S	0.712	0.285	-0.468	0.241
MDORS28	0.712	0.401	-0.589	0.353

CDORS15_S	0.622	0.199	-0.441	0.316
MDORS15_S	0.527	0.195	-0.462	0.392
MDORS14_S	0.428	0.078	-0.235	0.340
MDORS16_RS_S	0.243	0.794	-0.374	-0.011
MDORS8_RS	0.224	0.782	-0.291	0.098
MDORS10_RS	0.184	0.772	-0.304	0.065
MDORS18_RS_S	0.095	0.767	-0.239	-0.095
MDORS22_RS_S	0.368	0.748	-0.479	0.083
MDORS3_RS	0.361	0.725	-0.494	0.188
MDORS11_RS	0.207	0.557	-0.241	0.054
MDORS6_RS	0.244	0.542	-0.337	0.185
MDORS1_RS	0.131	0.523	-0.225	0.047
MDORS25	0.555	0.422	-0.887	0.344
MDORS27	0.556	0.391	-0.845	0.334
MDORS23	0.656	0.422	-0.777	0.397
MDORS21	0.460	0.402	-0.735	0.286
MDORS13	0.421	0.282	-0.721	0.310
MDORS17_S	0.424	0.070	-0.308	0.803
MDORS9_S	0.360	0.044	-0.279	0.767
MDORS20_S	0.414	0.227	-0.473	0.543
MDORS7_S	0.467	0.174	-0.401	0.515
MDORS12_S	0.459	0.148	-0.319	0.489

Note. Maximum Likelihood Extraction method was used in combination with a Direct Oblimin rotation with Kaiser normalization; item names indicate original source, whether item was reverse scored and response-option type.

Total Variance Explained

	Initial Eigenvalues			Extraction Sums of Squared Loadings			Rotation Sums of Squared Loadings[1]
Factor	Total	% of variance	Cumulative %	Total	% of variance	Cumulative %	Total
1	9.832	35.114	35.114	9.372	33.471	33.471	7.297
2	3.751	13.395	48.509	3.287	11.740	45.211	5.930
3	1.497	5.348	53.857	1.063	3.798	49.009	7.017
4	1.434	5.121	58.978	1.004	3.587	52.595	4.032
5	0.966	3.451	62.429				
6	0.961	3.432	65.862				
7	0.840	2.999	68.861				
8	0.790	2.820	71.681				
9	0.750	2.680	74.361				
10	0.660	2.357	76.719				
11	0.624	2.230	78.949				
12	0.564	2.016	80.965				
13	0.548	1.958	82.923				
14	0.501	1.791	84.714				

15	0.476	1.699	86.413				
16	0.443	1.582	87.995				
17	0.394	1.407	89.403				
18	0.364	1.299	90.702				
19	0.357	1.274	91.976				
20	0.317	1.132	93.108				
21	0.310	1.108	94.215				
22	0.294	1.050	95.266				
23	0.268	0.958	96.224				
24	0.241	0.859	97.083				
25	0.232	0.827	97.910				
26	0.211	0.752	98.663				
27	0.195	0.697	99.359				
28	0.179	0.641	100				

When factors are correlated, sums of squared loadings cannot be added to obtain a total variance

Component Correlation Matrix

Factor	1	2	3	4
1	1.000	0.301	-0.602	0.501
2		1.000	-0.446	0.082
3			1.000	-0.379

Analysis D: PCA - 12-item DORS with subjective response options, number of factors selected using Kaiser's criterion.

Total Variance Explained

	Initial Eigenvalues			Extraction Sums of Squared Loadings			Rotation Sums of Squared Loadings[1]
Factor	Total	% of variance	Cumulative %	Total	% of variance	Cumulative %	Total
1	4.625	38.543	38.543	4.239	35.326	35.326	3.389
2	2.211	18.429	56.971	1.874	15.614	50.940	2.335
3	1.190	9.919	66.891	0.884	7.368	58.308	3.297
4	1.081	9.012	75.902	0.776	6.471	64.779	2.405
5	0.606	5.052	80.955				
6	0.455	3.792	84.746				
7	0.437	3.645	88.391				
8	0.325	2.712	91.103				
9	0.317	2.640	93.743				
10	0.284	2.367	96.111				
11	0.251	2.088	98.199				
12	0.216	1.801	100.00				

Scree Plot

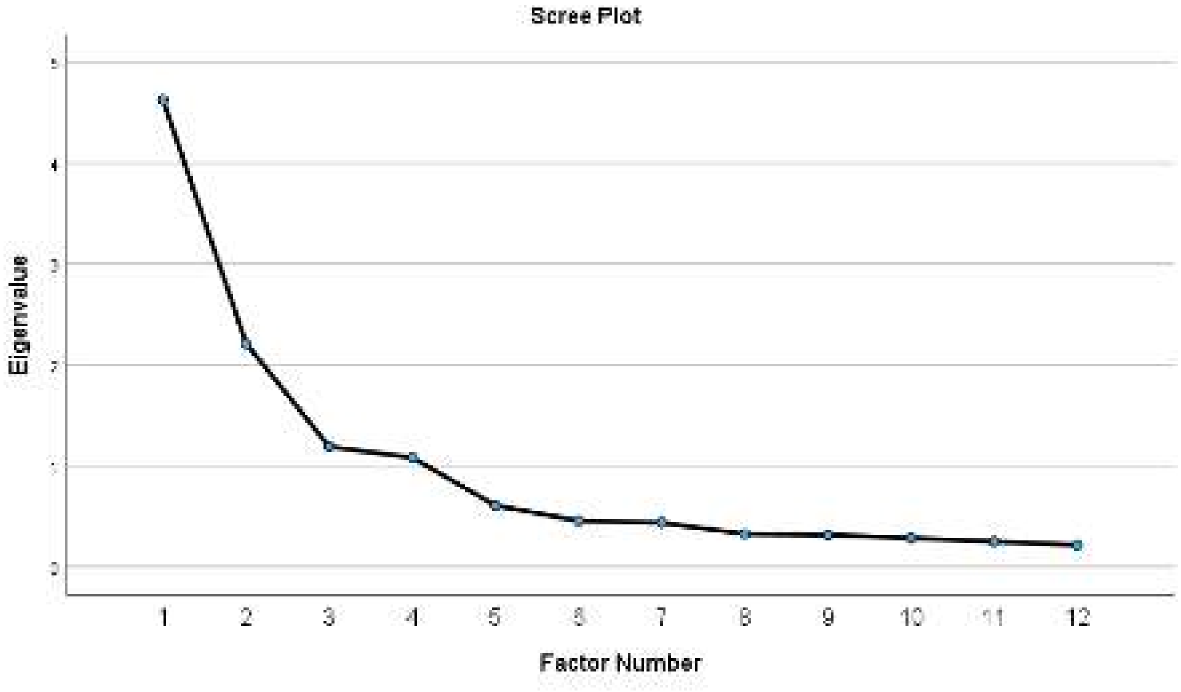

Parallel Analysis

Number of variables: 12, Number of subjects: 354, Number of replications: 100

Eigenvalue #	Initial Eigenvalue	Random Eigenvalue	Standard Deviation
1	**4.625**	1.3136	0.0423
2	**2.211**	1.2266	0.0304
3	**1.190**	1.1649	0.0252
4	1.081	1.1095	0.0228
5	0.606	1.0595	0.0241

Notes: truncated at 5 rows, bold type denotes where initial eigenvalue exceeds random eigenvalue. Generated using Monte Carlo PCA for Parallel Analysis, Version 3.0, ©2000-2020 by Marley W. Watkins. All rights reserved.

Pattern Matrix

Item Source	1 (EMR)	2 (PCO)	3 (AFF)	4 (ENG)
MDORS25	**0.94**	0.02	-0.026	0.004
MDORS27	**0.735**	0.031	0.112	-0.006
MDORS13	**0.725**	-0.018	0.004	0.01
MDORS8_RS	-0.073	**0.866**	0.055	0.078
MDORS10_RS	0.02	**0.817**	-0.011	0.024
MDORS18_RS_S	0.056	**0.737**	-0.039	-0.115
MDORS24_S	0.013	-0.003	**0.865**	0.01
MDORS4_S	-0.073	0.031	**0.805**	0.015
CDORS21_S	0.159	-0.036	**0.738**	-0.012
MDORS9_S	-0.014	-0.026	-0.061	**0.886**
MDORS17_S	-0.023	-0.012	0.113	**0.72**
MDORS20_S	0.28	0.082	0.039	**0.375**

Note. Maximum Likelihood Extraction method was used in combination with a Direct Oblimin rotation with Kaiser normalization; item names indicate original source, whether item was reverse scored and response-option type; bold text conveys loadings exceeding ± 0.3; rotation converged in 10 iterations.

Structure Matrix

Item Source	1	2	3	
MDORS25	0.934	0.340	0.505	0.349
MDORS27	0.806	0.305	0.525	0.329
MDORS13	0.725	0.234	0.410	0.287
MDORS8_RS	0.287	0.854	0.209	0.106
MDORS10_RS	0.305	0.822	0.159	0.053
MDORS18_RS_S	0.245	0.746	0.069	-0.089
MDORS24_S	0.498	0.158	0.876	0.440
CDORS21_S	0.554	0.151	0.814	0.409
MDORS4_S	0.392	0.151	0.777	0.384
MDORS9_S	0.279	-0.013	0.362	0.850
MDORS17_S	0.309	0.024	0.452	0.766
MDORS20_S	0.473	0.199	0.395	0.503

Note. Maximum Likelihood Extraction method was used in combination with a Direct Oblimin rotation with Kaiser normalization; item names indicate original source, whether item was reverse scored and response-option type.

Component Correlation Matrix

Factor	1 (EMR)	2 (PCO)	3 (AFF)	4 (ENG)
1	1.000	0.345	0.558	0.380
2		1.000	0.180	0.033
3			1.000	0.491
