# Supplementary material for: New 28-Item and 12-Item Dog Owner Relationship Scales: Contemporary Versions of the MDORS with a Revised Four-Component Structure
Source: Animals (Basel). 2025 Feb 21;15(5):632. doi: 10.3390/ani15050632 (PMC11898123; doi:10.3390/ani15050632)
Supplement: Supplementary file 1 [file animals-15-00632-s001.zip › File S4 - DORS28 and DORS12 subscale properties and relationships with other variables .pdf]

File S4: DORS28 and DORS12 subscale properties and relationships with other variables

DORS28 Descriptives

	DORS28-PCO	DORS28-AFF	DORS28-EMR	DORS28-ENG	DORS28-TOT
N	354	354	354	354	354
Missing	0	0	0	0	0
Mean	5.79	5.26	6.06	4.13	21.2
Median	6.11	5.33	6.40	4.20	21.7
Standard deviation	1.05	1.05	1.03	1.07	3.23
Minimum	2.00	1.78	1.00	1.40	7.24
Maximum	7.00	7.00	7.00	7.00	28.0
Skewness	-1.02	-0.705	-1.52	-0.0730	-0.798
Std. error skewness	0.130	0.130	0.130	0.130	0.130
Kurtosis	0.670	0.479	3.07	-0.358	1.08
Std. error kurtosis	0.259	0.259	0.259	0.259	0.259
Shapiro-Wilk W	0.906	0.964	0.841	0.993	0.965
Shapiro-Wilk p	< .001	< .001	< .001	0.112	< .001

DORS12 Descriptives

	DORS12-PCO	DORS12-AFF	DORS12-EMR	DORS12-ENG	DORS12-TOT
N	354	354	354	354	354
Missing	0	0	0	0	0
Mean	5.81	5.13	6.04	3.92	20.9
Median	6.33	5.33	6.33	4.00	21.3
Standard deviation	1.22	1.37	1.04	1.23	3.41
Minimum	1.33	1.00	1.00	1.00	8.00
Maximum	7.00	7.00	7.00	7.00	28.0
Skewness	-1.09	-0.765	-1.52	0.0134	-0.610
Std. error skewness	0.130	0.130	0.130	0.130	0.130
Kurtosis	0.582	0.280	2.95	-0.502	0.304
Std. error kurtosis	0.259	0.259	0.259	0.259	0.259
Shapiro-Wilk W	0.867	0.942	0.839	0.988	0.975
Shapiro-Wilk p	< .001	< .001	< .001	0.005	< .001

DORS28 - Histograms

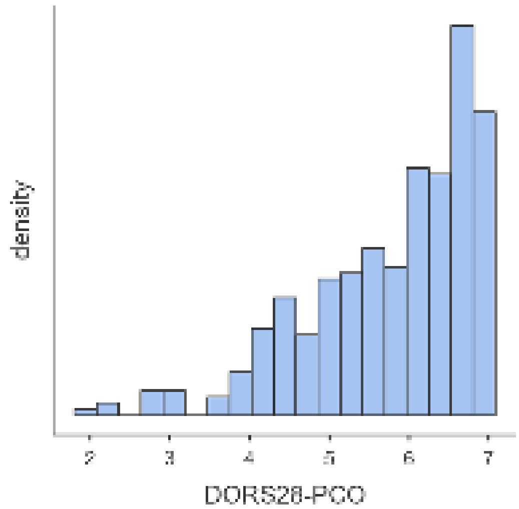

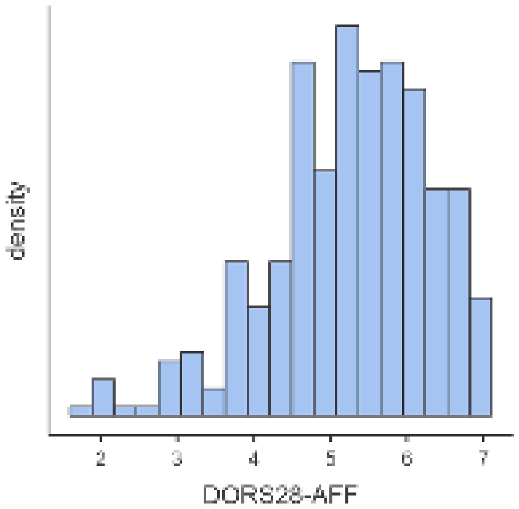

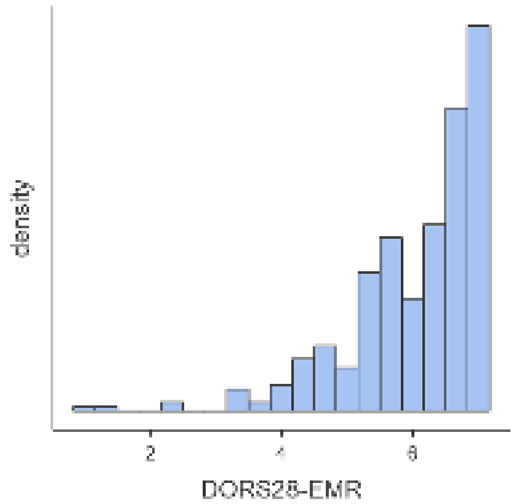

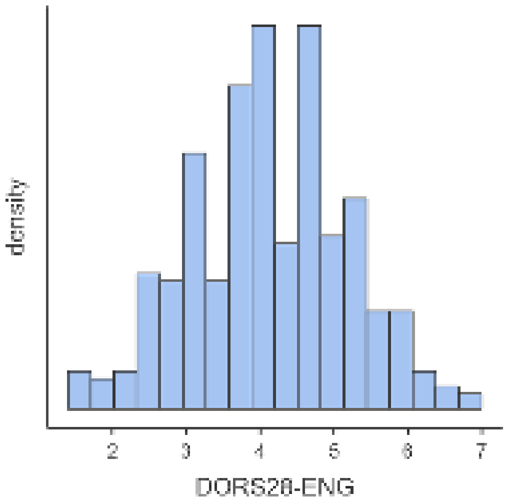

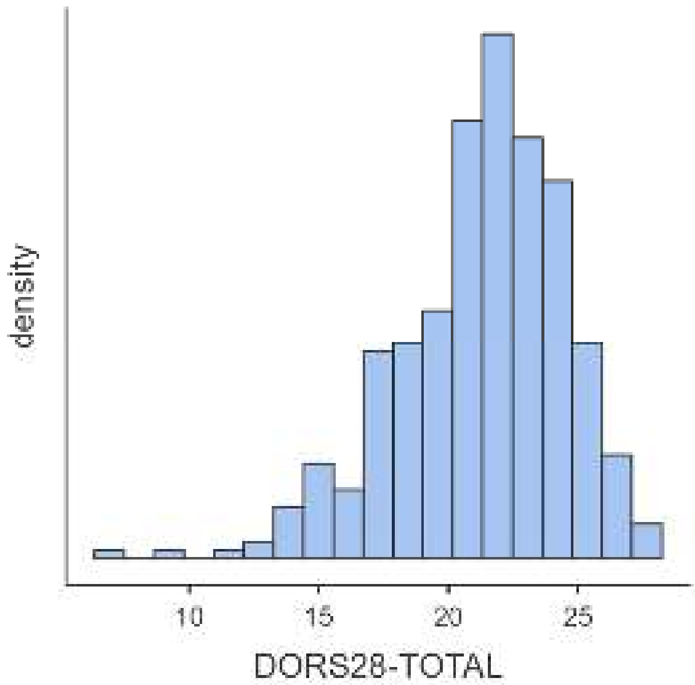

DORS12 - Histograms

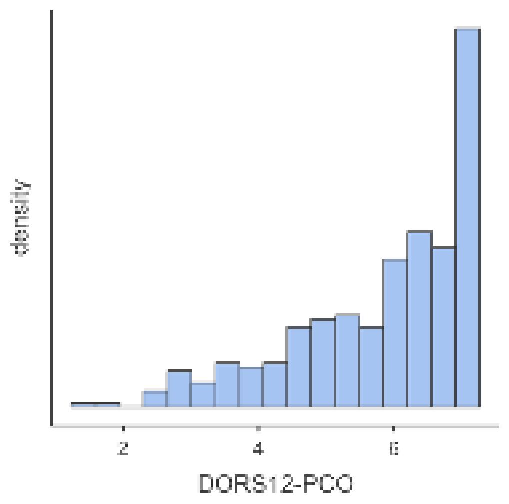

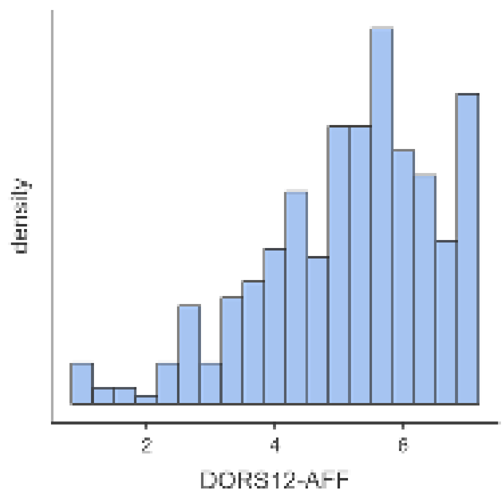

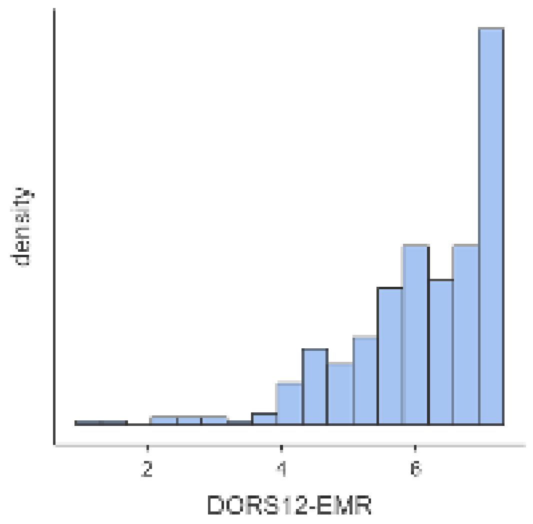

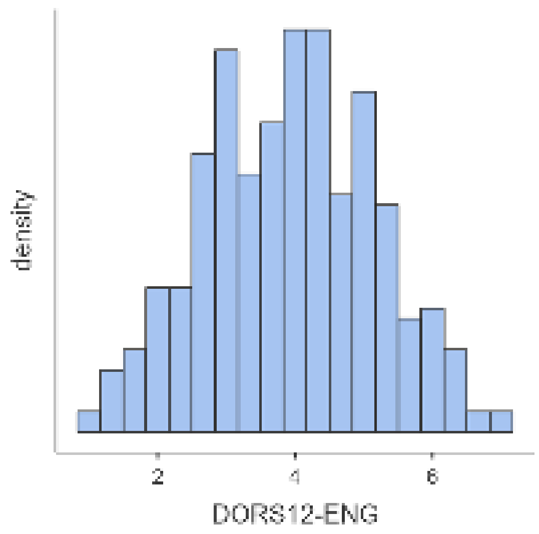

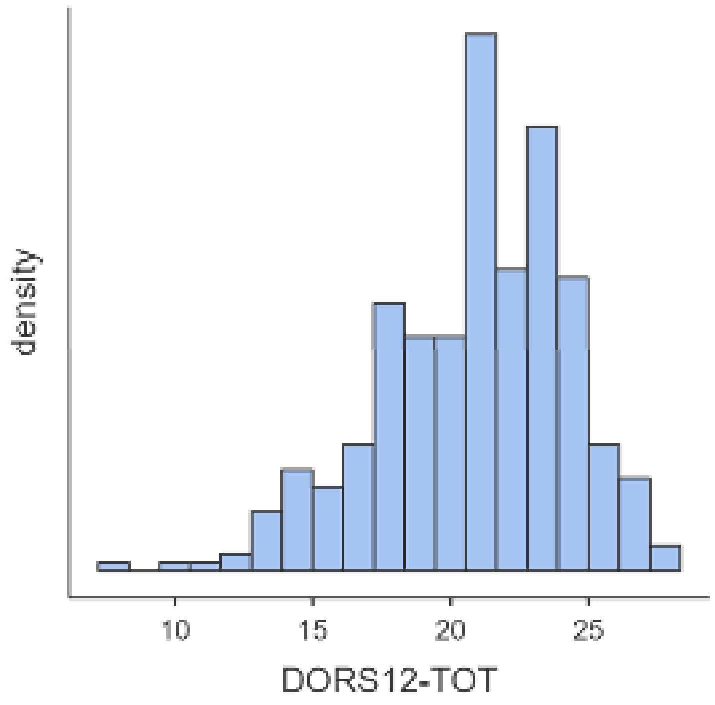

Correlation Matrix – DORS28 VS D0RS12 VS LAPS

		DORS28-PCO	DORS28-AFF	DORS28-EMR	DORS28-ENG	DORS28-TOT	DORS12-PCO	DORS12-AFF	DORS12-EMR	DORS12-ENG	DORS12-TOT
DORS28-AFF											
	Spearman's rho	0.371	—								
	df	352	—								
	p-value	< .001***	—								
DORS28-EMR											
	Spearman's rho	0.494	0.644	—							
	df	352	352	—							
	p-value	< .001***	< .001***	—							
DORS28-ENG											
	Spearman's rho	0.212	0.529	0.441	—						
	df	352	352	352	—						
	p-value	< .001***	< .001***	< .001***	—						
DORS28-TOT											
	Spearman's rho	0.67	0.81	0.822	0.713	—					
	df	352	352	352	352	—					
	p-value	< .001***	< .001***	< .001***	< .001***	—					
DORS12-PCO											
	Spearman's rho	0.883	0.308	0.41	0.127	0.563	—				
	df	352	352	352	352	352	—				
	p-value	< .001***	< .001***	< .001***	0.017*	< .001***	—				
DORS12-AFF											
	Spearman's rho	0.301	0.909	0.529	0.493	0.709	0.235	—			
	df	352	352	352	352	352	352	—			
	p-value	< .001***	< .001***	< .001***	< .001***	< .001***	< .001***	—			
DORS12-EMR											
	Spearman's rho	0.447	0.601	0.956	0.401	0.762	0.377	0.49	—		
	df	352	352	352	352	352	352	352	—		
	p-value	< .001***	< .001***	< .001***	< .001***	< .001***	< .001***	< .001***	—		
DORS12-ENG											
	Spearman's rho	0.165	0.455	0.391	0.934	0.638	0.092	0.425	0.355	—	
	df	352	352	352	352	352	352	352	352	—	

	p-value	0.002**	< .001***	< .001***	< .001***	< .001***	0.083	< .001***	< .001***	—	
DORS12-TOT											
	Spearman's rho	0.623	0.794	0.76	0.704	0.952	0.581	0.769	0.729	0.677	—
	df	352	352	352	352	352	352	352	352	352	—
	p-value	< .001***	< .001***	< .001***	< .001***	< .001***	< .001***	< .001***	< .001***	< .001***	—
LAPS-SS1											
	Spearman's rho	0.475	0.606	0.760	0.457	0.740	0.381	0.481	0.693	0.399	0.672
	df	350	350	350	350	350	350	350	350	350	350
	p-value	< .001***	< .001***	< .001***	< .001***	< .001***	< .001***	< .001***	< .001***	< .001***	< .001***
LAPS-SS2											
	Spearman's rho	0.424	0.613	0.731	0.445	0.716	0.337	0.501	0.654	0.419	0.653
	df	350	350	350	350	350	350	350	350	350	350
	p-value	< .001***	< .001***	< .001***	< .001***	< .001***	< .001***	< .001***	< .001***	< .001***	< .001***
LAPS-SS3											
	Spearman's rho	0.450	0.601	0.686	0.356	0.680	0.393	0.491	0.635	0.315	0.627
	df	350	350	350	350	350	350	350	350	350	350
	p-value	< .001***	< .001***	< .001***	< .001***	< .001***	< .001***	< .001***	< .001***	< .001***	< .001***
LAPS-TOTAL											
	Spearman's rho	0.493	0.662	0.800	0.465	0.782	0.403	0.535	0.727	0.419	0.714
	df	350	350	350	350	350	350	350	350	350	350
	p-value	< .001***	< .001***	< .001***	< .001***	< .001***	< .001***	< .001***	< .001***	< .001***	< .001***

Note. * p < .05, ** p < .01, *** p < .001

Correlation Matrix - DORS28 VS Demographic Variables

		DORS28-PCO	DORS28-AFF	DORS28-EMR	DORS28-ENG	DORS28-TOT
DogBehSatis						
	Spearman's rho	0.481	0.242	0.313	0.261	0.413
	df	352	352	352	352	352
	p-value	< .001***	< .001***	< .001***	< .001***	< .001***
DogHealthSatis						
	Spearman's rho	0.219	0.110	0.176	0.192	0.219
	df	352	352	352	352	352
	p-value	< .001***	0.039*	< .001***	< .001***	0.001**
DogR/POverall						
	Spearman's rho	0.385	0.480	0.530	0.431	0.586
	df	352	352	352	352	352
	p-value	< .001***	< .001***	< .001***	< .001***	< .001***
DogBond						
	Spearman's rho	0.352	0.535	0.664	0.388	0.614
	df	350	350	350	350	350
	p-value	< .001***	< .001***	< .001***	< .001***	< .001***
Age-participant						
	Spearman's rho	0.221	0.026	0.056	-0.003	0.091
	df	352	352	352	352	352
	p-value	< .001***	0.626	0.293	0.962	0.087
EducLev						
	Spearman's rho	-0.008	0.089	0.023	0.231	0.119
	df	352	352	352	352	352
	p-value	0.876	0.093	0.662	< .001***	0.025*
AdultsInHome						
	Spearman's rho	-0.089	-0.055	-0.174	-0.057	-0.136
	df	352	352	352	352	352
	p-value	0.093	0.305	0.001**	0.284	0.010*
ChildrenInHome						
	Spearman's rho	-0.126	-0.108	-0.160	-0.015	-0.147
	df	352	352	352	352	352
	p-value	0.018*	0.042*	0.002**	0.781	0.006**
CurrDogNumb						
	Spearman's rho	0.149	0.018	0.014	-0.121	0.010
	df	352	352	352	352	352
	p-value	0.005**	0.742	0.800	0.022*	0.846
DogAge						
	Spearman's rho	0.126	0.019	0.006	-0.217	-0.019
	df	352	352	352	352	352
	p-value	0.017*	0.728	0.909	< .001***	0.723
DogAgeAquire						
	Spearman's rho	0.027	0.058	0.055	-0.092	0.021
	df	352	352	352	352	352
	p-value	0.607	0.275	0.298	0.085	0.699
DogWeightPounds						
	Spearman's rho	-0.052	-0.011	0.079	0.007	0.011
	df	352	352	352	352	352
	p-value	0.330	0.831	0.136	0.895	0.839

Note. * p < .05, ** p < .01, *** p < .001

Analysis of group differences on the DORS28

DORS28 x Gender

	Gender-participant	DORS28-PCO	DORS28-AFF	DORS28-EMR	DORS28-ENG	DORS28-TOTAL
N	Female	217	217	217	217	217
	Male	134	134	134	134	134
Mean	Female	5.87	5.39	6.10	4.14	21.50
	Male	5.69	5.04	5.98	4.11	20.81
Median	Female	6.11	5.56	6.40	4.20	21.89
	Male	5.94	5.00	6.20	4.20	21.37
Standard deviation	Female	1.00	1.03	0.96	1.07	3.07
	Male	1.13	1.05	1.13	1.07	3.47
Shapiro-Wilk	W	0.913	0.966	0.854	0.994	0.968
	p	< .001***	<.001***	< .001***	0.190	< .001***
Levene's	F	5.4803	0.2018	1.2818	0.0336	2.0119
	df1	1	1	1	1	1
	df2	349	349	349	349	349
	p	0.020*	0.654	0.258	0.855	0.157
Independent Sample T-Test	Student's t				0.258	
	df				349	
	p				0.797	
	Mann-Whitney U	13407	11437	13877		13087
	p	0.220	< .001***	0.469		0.116

Note. * $p < .05$, ** $p < .01$, *** $p < .001$; Student's t assumes equal variances. Mann Whitney U was used for comparisons where Shapiro Wilk test was significant.

DORS28 X Employment Status

		DORS28-PCO	DORS28-AFF	DORS28-EMR	DORS28-ENG	DORS28-TOTAL
N	Full Time Paid Work	193	193	193	193	193
	Unemployed	28	28	28	28	28
	Home Duties	21	21	21	21	21
	Unable to work	13	13	13	13	13
	Part time Paid Work	58	58	58	58	58
	Retired	17	17	17	17	17
	Student	15	15	15	15	15
	Other	5	5	5	5	5
	Prefer Not to Say	4	4	4	4	4
Mean	Full Time Paid Work	5.81	5.32	6.12	4.31	21.6
	Unemployed	5.87	5.08	5.93	3.74	20.6
	Home Duties	5.41	4.93	5.55	3.47	19.4
	Unable to work	5.95	4.84	5.55	3.12	21.5
	Part time Paid Work	5.85	5.28	6.11	4.24	216.5
	Retired	6.08	5.38	6.27	3.96	21.7

	Student	5.39	5.38	6.04	4.12	20.9
	Other	5.89	5.24	6.40	4.16	21.7
	Prefer Not to Say	5.47	5.81	6.15	4.20	21.6
Median	Full Time Paid Work	6.11	5.44	6.40	4.20	21.8
	Unemployed	6.22	5.44	6.40	3.80	21.2
	Home Duties	5.89	5.22	5.80	3.40	20.7
	Unable to work	6.22	4.67	5.80	2.80	18.4
	Part time Paid Work	6.28	5.33	6.40	4.40	22.0
	Retired	6.33	5.00	6.40	3.80	22.0
	Student	5.33	5.33	6.20	4.00	21.1
	Other	6.00	5.11	6.40	4.20	21.6
	Prefer Not to Say	5.56	5.67	6.20	4.10	22.0
Standard deviation	Full Time Paid Work	1.072	0.974	0.931	1.032	3.030
	Unemployed	1.010	1.360	1.377	1.106	3.978
	Home Duties	1.498	1.416	1.420	1.061	4.414
	Unable to work	0.899	1.651	1.573	1.145	4.513
	Part time Paid Work	0.941	0.989	0.951	1.029	2.966
	Retired	0.945	0.871	0.784	0.828	2.625
	Student	0.899	0.673	0.772	0.970	2.284
	Other	0.444	0.288	0.490	0.518	0.253
	Prefer Not to Say	1.149	0.833	0.806	1.356	2.904
Shapiro-Wilk	W	0.919	0.977	0.886	0.995	0.975
	P	< .001***	< .001***	< .001***	0.236	< .001***
Levene's	F	1.614	4.104	3.053	0.758	3.408
	df1	8	8	8	8	8
	df2	345	345	345	345	345
	p	0.119	< .001***	0.002**	0.640	0.001**
One-Way Anova (Fisher's)	F				4.01	
	df1				8	
	df2				345	
	p				<.001***	
Kruskal Wallis	χ^2	6.51	2.64	5.68		5.72
	df	8	8	8		8
	p	0.590	0.955	0.683		0.679
	ε^2	0.01845	0.00747	0.01610		0.02153

Note. * p < .05, ** p < .01, *** p < .001; Fisher's test assumes equal variances, Kruskal-Wallis was used for comparisons where Shapiro Wilk test was significant.

Tukey Post-Hoc Test – DORS28-ENG

		Un-employed	Home duties	Unable to work	Part time paid Work	Retired	Student	Other	Prefer not to Say
Full time paid work	Mean diff.	0.567	0.843	1.187	0.0650	0.345	0.190	0.1498	0.1098
	p-value	0.145	0.012*	0.002**	1.000	0.924	0.999	1.000	1.000
Un-employed	Mean diff.	—	0.276	0.620	-0.5020	-0.222	-0.377	-0.4171	-0.4571
	p-value	—	0.991	0.688	0.464	0.999	0.967	0.996	0.996
Home duties	Mean diff.		—	0.344	-0.7782	-0.498	-0.653	-0.6933	-0.7333
	p-value		—	0.990	0.078	0.864	0.632	0.915	0.930
Unable to work	Mean diff.			—	-1.1218	-0.842	-0.997	-1.0369	-1.0769
	p-value			—	0.013*	0.398	0.211	0.606	0.664
Part time paid Work	Mean diff.				—	0.280	0.125	0.0848	0.0448
	p-value				—	0.987	1.000	1.000	1.000
Retired	Mean diff.					—	-0.155	-0.1953	-0.2353
	p-value					—	1.000	1.000	1.000
Student	Mean diff.						—	-0.0400	-0.0800
	p-value						—	1.000	1.000
Other	Mean diff.							—	-0.0400
	p-value							—	1.000

Note. * $p < .05$, ** $p < .01$, *** $p < .001$; Tukey post hoc tests assume equal variances and was used for pairwise comparisons for subscales where ANOVA revealed significant group differences.

DORS28 X Marital Status

		DORS28-PCO	DORS28-AFF	DORS28-EMR	DORS28-ENG	DORS28-TOTAL
N	Separated/Divorced/Widowed	41	41	41	41	41
	Married	153	153	153	153	153
	Single/Never Married	118	118	118	118	118
	Defacto/Living together	36	36	36	36	36
	Prefer Not to Say	6	6	6	6	6
Mean	Separated/Divorced/Widowed	6.25	5.47	6.26	4.15	22.1
	Married	5.66	5.16	5.92	4.17	20.9
	Single/Never Married	5.75	5.25	6.11	4.01	21.1
	Defacto/Living together	6.12	5.60	6.38	4.42	22.5
	Prefer Not to Say	5.06	4.83	5.03	3.80	18.7
Median	Separated/Divorced/Widowed	6.44	5.33	6.60	4.00	22.2
	Married	5.89	5.33	6.00	4.40	21.4
	Single/Never Married	6.00	5.33	6.50	4.00	21.6
	Defacto/Living together	6.22	5.56	6.80	4.20	22.6
	Prefer Not to Say	4.72	5.06	4.70	3.80	17.6

Standard deviation	Separated/Divorced/Widowed	0.768	1.06	0.951	1.02	3.13
	Married	1.12	1.10	1.00	1.08	3.25
	Single/Never Married	1.06	0.990	1.09	1.05	3.16
	Defacto/Living together	0.789	0.836	0.796	1.02	2.84
	Prefer Not to Say	1.13	1.62	1.21	1.40	4.22
Shapiro-Wilk	W	0.930	0.970	0.870	0.993	0.967
	P	< .001***	< .001***	< .001***	0.097	< .001***
Levene's	F	3.622	1.776	0.765	0.683	1.152
	df1	4	4	4	4	4
	df2	349	349	349	349	349
	p	0.007**	0.133	0.549	0.604	0.332
One-Way Anova (Fisher's)	F				1.28	
	df1				4	
	df2				349	
	p				0.279	
Kruskal Wallis	χ^2	16.35	5.40	16.44		12.85
	df	4	4	4		4
	p	0.003**	0.249	0.002**		0.012*
	ε^2	0.0463	0.0153	0.0466		0.0364

Note. * p < .05, ** p < .01, *** p < .001; Fisher's test assumes equal variances, Kruskal-Wallis was used for comparisons where Shapiro Wilk test was significant.

Dwass-Steel-Critchlow-Fligner Pairwise comparisons

DORS28-PCO			
		W	p
Separated/Divorced/Widowed	Married	-4.576	**0.011***
	Single/Never Married	-3.728	0.064
	Defacto/Living together	-1.139	0.929
	Prefer Not to Say	-3.419	0.111
Married	Single/Never Married	0.814	0.979
	Defacto/Living together	3.174	0.164
	Prefer Not to Say	-1.900	0.664
Single/Never Married	Defacto/Living together	2.374	0.447
	Prefer Not to Say	-2.326	0.469
Defacto/Living together	Prefer Not to Say	-3.189	0.160
**DORS28-EMR**			
		W	p
Separated/Divorced/Widowed	Married	-3.460	0.103
	Single/Never Married	-1.566	0.803
	Defacto/Living together	-0.111	1.000
	Prefer Not to Say	-3.231	0.150
Married	Single/Never Married	3.008	0.209
	Defacto/Living together	3.828	0.053
	Prefer Not to Say	-2.711	0.308
Single/Never Married	Defacto/Living together	1.757	0.727

	Prefer Not to Say	-2.999	0.211
Defacto/Living together	Prefer Not to Say	-3.341	0.126
DORS28-TOT			
		W	p
Separated/Divorced/Widowed	Married	-2.919	0.236
	Single/Never Married	-2.205	0.524
	Defacto/Living together	0.953	0.962
	Prefer Not to Say	-2.615	0.345
Married	Single/Never Married	0.975	0.959
	Defacto/Living together	3.843	0.051
	Prefer Not to Say	-1.905	0.662
Single/Never Married	Defacto/Living together	3.493	0.098
	Prefer Not to Say	-1.935	0.648
Defacto/Living together	Prefer Not to Say	-2.694	0.315

Note. * p < .05, ** p < .01, *** p < .001; Used for pairwise comparisons for subscales where Kruskal-Wallis revealed significant group differences.

DORS28 X Home Location

		DORS28-PCO	DORS28-AFF	DORS28-EMR	DORS28-ENG	DORS28-TOTAL
N	Suburban	165	165	165	165	165
	Regional City	27	27	27	27	27
	Rural	49	49	49	49	49
	Urban	73	73	73	73	73
	Country Town	40	40	40	40	40
Mean	Suburban	5.77	5.34	6.02	4.10	21.2
	Regional City	5.76	5.07	6.07	3.90	20.8
	Rural	5.95	5.05	5.82	3.97	20.8
	Urban	5.68	5.36	6.22	4.48	21.7
	Country Town	5.91	5.18	6.18	4.00	21.3
Median	Suburban	6.00	5.56	6.20	4.00	21.7
	Regional City	6.22	5.33	6.80	4.20	21.3
	Rural	6.33	5.22	6.00	4.00	21.3
	Urban	5.89	5.44	6.40	4.60	21.8
	Country Town	6.33	5.33	6.40	3.80	21.9
Standard deviation	Suburban	1.05	0.983	1.01	0.980	3.10
	Regional City	1.08	1.09	1.44	1.19	3.78
	Rural	0.948	1.31	1.24	1.24	3.85
	Urban	1.05	0.907	0.778	0.984	2.59
	Country Town	1.18	1.18	0.838	1.14	3.63
Shapiro-Wilk	W	0.913	0.971	0.872	0.996	0.968

	P	< .001***	< .001***	< .001***	0.403	< .001***
Levene's	F	0.378	2.132	3.386	2.800	2.178
	df1	4	4	4	4	4
	df2	349	349	349	349	349
	p	0.824	0.076	0.010*	0.026*	0.071
One-Way Anova (Welch's)	F				2.84	
	df1				4	
	df2				97.8	
	p				0.028*	
Kruskal Wallis	χ^2	3.54	2.74	3.47		1.97
	df	4	4	4		4
	p	0.471	0.603	0.482		0.741
	ε^2	0.01004	0.01054	0.00984		0.00810

Note. * p < .05, ** p < .01, *** p < .001; Welch's test does not assume equal variances, Kruskal-Wallis was used for comparisons where Shapiro Wilk test was significant.

Games-Howell Post-Hoc Test – DORS28-ENG

		Suburban	Regional City	Rural	Urban	Country Town
Suburban	Mean difference	—	0.196	0.1320	-0.380	0.1044
	p-value	—	0.925	0.959	0.052	0.984
Regional City	Mean difference		—	-0.0636	-0.576	-0.0913
	p-value		—	0.999	0.183	0.998
Rural	Mean difference			—	-0.512	-0.0277
	p-value			—	0.118	1.000
Urban	Mean difference				—	0.4845
	p-value				—	0.171
Country Town	Mean difference					—
	p-value					—

Note. * p < .05, ** p < .01, *** p < .001; Games-Howell is used for post hoc tests when variances are unequal.

DORS28 X Dwelling Type

		DORS28-PCO	DORS28-AFF	DORS28-EMR	DORS28-ENG	DORS28-TOTAL
N	House	269	269	269	269	269
	Unit/Apartment	61	61	61	61	61
	Semi-detached	21	21	21	21	21
	Trailer/RV	3	3	3	3	3
Mean	House	5.83	5.24	6.02	4.09	21.2
	Unit/Apartment	5.76	5.32	6.20	4.16	21.4
	Semi-detached	5.35	5.36	5.97	4.56	21.2
	Trailer/RV	6.59	5.96	6.93	4.33	23.8
Median	House	6.11	5.33	6.20	4.00	21.6

	Unit/Apartment	6.11	5.44	6.60	4.20	21.6
	Semi-detached	5.78	5.56	6.20	4.80	21.9
	Trailer/RV	6.56	6.00	7.00	4.60	22.8
Standard deviation	House	1.05	1.09	1.05	1.09	3.37
	Unit/Apartment	0.990	0.921	0.905	0.982	2.66
	Semi-detached	1.26	0.926	1.11	0.965	3.11
	Trailer/RV	0.390	0.834	0.115	1.03	1.87
Shapiro-Wilk	W	0.919	0.966	0.854	0.995	0.967
	P	< .001***	< .001***	< .001***	0.247	< .001***
Levene's	F	1.815	1.350	1.308	0.545	1.795
	df1	3	3	3	3	3
	df2	350	350	350	350	350
	p	0.144	0.258	0.271	0.652	0.148
One-Way Anova (Fisher's)	F				1.33	
	df1				3	
	df2				350	
	p				0.265	
Kruskal Wallis	χ^2	5.22	1.81	4.95		2.47
	df	3	3	3		3
	p	0.157	0.613	0.175		0.480
	ε^2	0.01478	0.00513	0.01403		0.00700

Note. * p < .05, ** p < .01, *** p < .001; Fisher's test assumes equal variances, Kruskal-Wallis was used for comparisons where Shapiro Wilk test was significant.

DORS28 X Dwelling Outside Space

		DORS28-PCO	DORS28-AFF	DORS28-EMR	DORS28-ENG	DORS28-TOTAL
N	None	13	13	13	13	13
	Small	128	128	128	128	128
	Medium	175	175	175	175	175
	Large	38	38	38	38	38
Mean	None	5.76	5.71	6.23	4.40	22.1
	Small	5.82	5.25	6.13	4.07	21.3
	Medium	5.73	5.22	6.00	4.15	21.1
	Large	5.98	5.35	6.00	4.19	21.5
Median	None	6.11	5.56	6.40	4.60	21.9
	Small	6.06	5.33	6.30	4.00	21.7
	Medium	6.00	5.33	6.40	4.20	21.5
	Large	6.44	5.39	6.40	4.50	22.1
Standard deviation	None	1.12	0.540	0.795	0.864	2.35
	Small	0.987	0.997	0.932	0.997	3.00
	Medium	1.10	1.10	1.10	1.09	3.45
	Large	1.04	1.14	1.07	1.24	3.25
Shapiro-Wilk	W	0.910	0.967	0.854	0.996	0.967
	P	< .001***	< .001***	< .001***	0.413	< .001***
Levene's	F	0.605	2.440	1.352	1.900	1.905

	df1	3	3	3	3	3
	df2	350	350	350	350	350
	p	0.612	0.064	0.257	0.129	0.128
One-Way Anova (Fisher's)	F				0.477	
	df2				3	
	df1				350	
	p				0.699	
Kruskal Wallis	χ^2	2.248	2.874	0.829		1.330
	df	3	3	3		3
	p	0.523	0.412	0.842		0.722
	ε^2	0.00637	0.00814	0.00235		0.00377

Note. * p < .05, ** p < .01, *** p < .001; Fisher's test assumes equal variances, Kruskal-Wallis was used for comparisons where Shapiro Wilk test was significant.

DORS28 X Dog Spend Time

		DORS28-PCO	DORS28-AFF	DORS28-EMR	DORS28-ENG	DORS28-TOTAL
N	Inside the house	251	251	251	251	251
	Both inside and outside equally	91	91	91	91	91
	Outside the house	12	12	12	12	12
Mean	Inside the house	5.83	5.28	6.09	4.07	21.3
	Both inside and outside equally	5.78	5.32	6.04	4.39	21.5
	Outside the house	5.06	4.49	5.47	3.50	18.5
Median	Inside the house	6.11	5.33	6.40	4.00	21.8
	Both inside and outside equally	6.22	5.44	6.40	4.60	21.6
	Outside the house	4.89	4.22	5.50	3.70	18.8
Standard deviation	Inside the house	1.01	1.05	1.01	1.07	3.18
	Both inside and outside equally	1.10	0.979	1.05	0.974	3.20
	Outside the house	1.29	1.38	1.15	1.18	3.59
Shapiro-Wilk	W	0.919	0.970	0.855	0.995	0.967
	P	< .001***	< .001***	< .001***	0.319	< .001***
Levene's	F	1.9490	2.097	0.3333	0.4562	0.361
	df1	2	2	2	2	2
	df2	351	351	351	351	351
	p	0.144	0.124	0.717	0.634	0.697
One-Way Anova (Fisher's)	F				5.37	
	df1				2	
	df2				351	

	p				0.005**	
Kruskal Wallis	χ^2	4.52	4.21	4.44		8.20
	df	2	2	2		2
	p	0.104	0.122	0.108		0.017*
	ε^2	0.01280	0.0119	0.01258		0.0232

Note. * p < .05, ** p < .01, *** p < .001; Fisher's test assumes equal variances, Kruskal-Wallis was used for comparisons where Shapiro Wilk test was significant.

Dwass-Steel-Critchlow-Fligner Pairwise comparisons

DORS28-TOT			
		**W**	**p**
Inside the house	Both inside and outside equally	0.938	0.785
Inside the house	Outside the house	-3.925	0.015*
Both inside and outside equally	Outside the house	-3.751	0.022*

Note. * p < .05, ** p < .01, *** p < .001; Used for pairwise comparisons for subscales where Kruskal-Wallis revealed significant group differences.

Tukey Post-Hoc Test – DORS28-ENG

		Inside the house	Both inside and outside equally	Outside the house
Inside the house	Mean difference	—	-0.322	0.569
	p-value	—	0.034*	0.161
Both inside and outside equally	Mean difference		—	0.891
	p-value		—	0.017*
Outside the house	Mean difference			—
	p-value			—

Note. * p < .05, ** p < .01, *** p < .001; Tukey's test assumes equal variances and was used when Fisher's test was significant.

DORS28 X Dog Sex

		DORS28-PCO	DORS28-AFF	DORS28-EMR	DORS28-ENG	DORS28-TOTAL
N	Male Entire	62	62	62	62	62
	Male Desexed	130	130	130	130	130
	Female Entire	40	40	40	40	40
	Female Desexed	120	120	120	120	120
Mean	Male Entire	5.63	5.16	6.10	4.42	21.3
	Male Desexed	5.80	5.28	6.11	4.06	21.2
	Female Entire	5.77	5.16	5.98	4.28	21.2
	Female Desexed	5.90	5.34	6.02	4.01	21.3
Median	Male Entire	5.89	5.33	6.40	4.70	21.8
	Male Desexed	6.11	5.33	6.40	4.10	21.9
	Female Entire	6.22	5.56	6.10	4.20	21.8

	Female Desexed	6.11	5.44	6.40	4.00	21.4
Standard deviation	Male Entire	1.19	1.04	0.993	1.21	3.42
	Male Desexed	1.000	0.927	0.969	0.961	2.91
	Female Entire	1.14	1.36	1.05	1.14	3.70
	Female Desexed	0.997	1.08	1.10	1.06	3.34
Shapiro-Wilk	W	0.915	0.966	0.851	0.994	0.965
	P	< .001***	< .001***	< .001***	0.223	< .001***
Levene's	F	1.429	3.956	0.626	2.358	1.401
	df1	3	3	3	3	3
	df2	348	348	348	348	348
	p	0.234	0.009**	0.599	0.072	0.242
One-Way Anova (Fisher's)	F				2.49	
	df1				3	
	df2				348	
	p				0.060	
Kruskal Wallis	χ^2	2.018	1.584	0.292		0.079
	df	3	3	3		3
	p	0.569	0.663	0.962		0.994
	ε^2	0.00575	0.00451	8.32e-4		2.261e-4

Note. * p < .05, ** p < .01, *** p < .001; Fisher's test assumes equal variances, Kruskal-Wallis was used for comparisons where Shapiro Wilk test was significant.

DORS28 X Dog Source

		DORS28-PCO	DORS28-AFF	DORS28-EMR	DORS28-ENG	DORS28-TOTAL
N	Pet Shop	14	14	14	14	14
	Breeder	99	99	99	99	99
	Shelter/Rescue	95	95	95	95	95
	From a Friend/Family Member	101	101	101	101	101
	Other	9	9	9	9	9
	Found	15	15	15	15	15
	Bred Myself	10	10	10	10	10
	Gift	6	6	6	6	6
	Inherited	5	5	5	5	5
Mean	Pet Shop	5.37	5.09	5.70	4.40	20.6
	Breeder	5.83	5.44	6.07	4.38	21.7
	Shelter/Rescue	5.97	5.32	6.23	4.06	21.6
	From a Friend/Family Member	5.69	5.16	5.98	4.03	20.9
	Other	5.79	5.53	6.22	3.96	21.5
	Found	5.84	5.10	6.17	3.96	21.1
	Bred Myself	5.56	4.76	5.62	4.06	20.0
	Gift	5.02	4.39	4.70	3.93	18.0
	Inherited	6.09	5.40	6.68	3.28	21.4
Median	Pet Shop	5.17	5.17	5.70	4.30	20.7

	Breeder	6.11	5.56	6.20	4.40	22.1
	Shelter/Rescue	6.33	5.33	6.40	4.00	22.2
	From a Friend/Family Member	6.00	5.11	6.20	4.00	21.3
	Other	6.00	5.56	6.80	4.40	21.9
	Found	6.22	5.56	6.80	4.20	23.0
	Bred Myself	5.39	4.56	5.60	4.30	20.2
	Gift	4.89	4.72	5.30	4.10	19.5
	Inherited	6.00	5.33	6.80	3.20	21.5
Standard deviation	Pet Shop	0.941	1.01	1.16	0.911	3.21
	Breeder	1.02	1.04	1.02	1.05	3.34
	Shelter/Rescue	1.01	1.00	0.810	0.944	2.88
	From a Friend/Family Member	1.13	1.07	0.979	1.15	3.17
	Other	1.01	0.769	0.997	1.08	2.70
	Found	1.26	1.36	1.40	1.22	4.20
	Bred Myself	0.726	1.06	1.13	1.24	3.26
	Gift	1.10	1.32	2.33	1.22	5.33
	Inherited	0.673	0.365	0.363	0.522	0.810
Shapiro-Wilk	W	0.918	0.969	0.901	0.996	0.969
	P	< .001***	< .001***	< .001***	0.487	< .001***
Levene's	F	0.784	1.118	4.014	1.457	1.519
	df1	8	8	8	8	8
	df2	345	345	345	345	345
	p	0.617	0.350	< .001***	0.172	0.149
One-Way Anova (Fisher's)	F				1.49	
	df1				8	
	df2				345	
	p				0.160	
Kruskal Wallis	χ^2	12.58	11.9	13.76		11.3
	df	8	8	8		8
	p	0.127	0.156	0.088		0.186
	ε^2	0.0356	0.0337	0.0390		0.0320

Note. * p < .05, ** p < .01, *** p < .001; Fisher's test assumes equal variances, Kruskal-Wallis was used for comparisons where Shapiro Wilk test was significant.

DORS28 X Dog Sports

	Active in Dog Sports	DORS28-PCO	DORS28-AFF	DORS28-EMR	DORS28-ENG	DORS28-TOTAL
N	No	270	270	270	270	270
	Yes	84	84	84	84	84
Mean	No	5.79	5.20	6.02	3.94	20.9
	Yes	5.81	5.48	6.18	4.75	22.2
Median	No	6.00	5.33	6.40	4.00	21.5
	Yes	6.22	5.56	6.40	4.90	22.5

Standard deviation	No	1.02	1.07	1.08	1.01	3.16
	Yes	1.18	0.970	0.841	0.998	3.28
Shapiro-Wilk	W	0.906	0.967	0.849	0.989	0.959
	P	< .001***	< .001***	< .001***	0.012*	< .001***
Levene's	F	4.19814	0.81539	2.81559	0.00303	1.04781
	df1	1	1	1	1	1
	df2	352	352	352	352	352
	p	0.041*	0.367	0.094	0.956	0.307
Independent Samples T-Test	Mann-Whitney U	10607	9630	10498	6508	8815
	p	0.370	0.037*	0.299	<.001***	0.002**

Note. * p < .05, ** p < .01, *** p < .001; Mann Whitney U was used for comparisons where Shapiro Wilk test was significant.

DORS28 X Dog Obedience

	Active in Dog Obedience	DORS28-PCO	DORS28-AFF	DORS28-EMR	DORS28-ENG	DORS28-TOTAL
N	No	245	245	245	245	245
	Yes	109	109	109	109	109
Mean	No	5.84	5.18	6.00	3.93	20.9
	Yes	5.69	5.46	6.19	4.59	21.9
Median	No	6.11	5.33	6.40	4.00	21.5
	Yes	6.11	5.56	6.40	4.80	22.4
Standard deviation	No	0.987	1.09	1.09	1.06	3.33
	Yes	1.19	0.924	0.852	0.934	2.89
Shapiro-Wilk	W	0.915	0.969	0.853	0.995	0.967
	P	< .001***	< .001***	< .001***	0.389	< .001***
Levene's	F	9.27	3.88	3.94	2.20	0.74
	df1	1	1	1	1	1
	df2	352	352	352	352	352
	p	0.003**	0.050	0.048*	0.139	0.39
Independent Samples T-Test	Student's t				-5.58	
	df				352	
	p				< .001***	
	Mann-Whitney U	12927	11427	12209		1107
	p	0.632	0.030*	0.194		0.009**

Note. * p < .05, ** p < .01, *** p < .001; Student's t assumes equal variances, Mann Whitney U was used for comparisons where Shapiro Wilk test was significant.

DORS28 X Dog Tricks

	Active in Dog Trick Training	DORS28-PCO	DORS28-AFF	DORS28-EMR	DORS28-ENG	DORS28-TOTAL
N	No	233	233	233	233	233
	Yes	121	121	121	121	121
Mean	No	5.78	5.14	5.95	4.01	20.9
	Yes	5.82	5.51	6.26	4.38	22.0
Median	No	6.00	5.22	6.20	4.00	21.3
	Yes	6.22	5.56	6.60	4.40	22.2
Standard deviation	No	1.02	1.11	1.10	1.10	3.35
	Yes	1.11	0.882	0.839	0.943	2.87
Shapiro-Wilk	W	0.907	0.971	0.866	0.995	0.968
	P	< .001***	< .001***	< .001***	0.258	< .001***
Levene's	F	1.23	6.42	7.49	4.86	3.69
	df1	1	1	1	1	1
	df2	352	352	352	352	352
	p	0.269	0.012*	0.007**	0.028*	0.056
Independent Samples T-Test	Welch's t test				-3.123	
	df				279	
	p				0.001**	
	Mann-Whitney U	13357	11368	11717		11289
	p	0.418	0.003**	0.008**		0.002**

Note. * p < .05, ** p < .01, *** p < .001; Welch's test was used when normality was assumed but variances were unequal, Mann Whitney U was used for comparisons where Shapiro Wilk test was significant.
