# Supplementary material for: New 28-Item and 12-Item Dog Owner Relationship Scales: Contemporary Versions of the MDORS with a Revised Four-Component Structure
Source: Animals (Basel). 2025 Feb 21;15(5):632. doi: 10.3390/ani15050632 (PMC11898123; doi:10.3390/ani15050632)
Supplement: Supplementary file 1 [file animals-15-00632-s001.zip › File S5 - DORS28 and DORS12 administration and scoring instructions.pdf]

File S5: DORS28 and DORS12 administration and scoring instructions

**Dog Owner Relationship Scale**

Instructions: Please consider each of the following statements and indicate which option best describes how you feel or act. We are interested in your opinions. There are no correct or incorrect responses.

---

**1. How often does your dog stop you doing things you want to?**

| Never | Almost never | Occasionally | More than occasionally | Often | Very often | Extremely often |
|---|---|---|---|---|---|---|
| ☐ | ☐ | ☐ | ☐ | ☐ | ☐ | ☐ |

**2. How often do you kiss your dog?**

| Never | Almost never | Occasionally | More than occasionally | Often | Very often | Extremely often |
|---|---|---|---|---|---|---|
| ☐ | ☐ | ☐ | ☐ | ☐ | ☐ | ☐ |

**3. My dog provides me with constant companionship.**

| Strongly disagree | Disagree | Somewhat disagree | Neither agree nor disagree | Somewhat agree | Agree | Strongly agree |
|---|---|---|---|---|---|---|
| ☐ | ☐ | ☐ | ☐ | ☐ | ☐ | ☐ |

**4. How often do you take your dog in the car, on your bike, or on public transport?**

| Never | Almost never | Occasionally | More than occasionally | Often | Very often | Extremely often |
|---|---|---|---|---|---|---|
| ☐ | ☐ | ☐ | ☐ | ☐ | ☐ | ☐ |

**5. It bothers me that my dog stops me doing things I enjoyed before I owned it.**

| Strongly disagree | Disagree | Somewhat disagree | Neither agree nor disagree | Somewhat agree | Agree | Strongly agree |
|---|---|---|---|---|---|---|
| ☐ | ☐ | ☐ | ☐ | ☐ | ☐ | ☐ |

**6. How often do you hug your dog?**

| Never | Almost never | Occasionally | More than occasionally | Often | Very often | Extremely often |
|---|---|---|---|---|---|---|
| ☐ | ☐ | ☐ | ☐ | ☐ | ☐ | ☐ |

**7. My dog is there whenever I need to be comforted.**

| Strongly disagree | Disagree | Somewhat disagree | Neither agree nor disagree | Somewhat agree | Agree | Strongly agree |
|---|---|---|---|---|---|---|
| ☐ | ☐ | ☐ | ☐ | ☐ | ☐ | ☐ |

**8. How often do you take your dog to visit people?**

| Never | Almost never | Occasionally | More than occasionally | Often | Very often | Extremely often |
|---|---|---|---|---|---|---|
| ☐ | ☐ | ☐ | ☐ | ☐ | ☐ | ☐ |

9. **It is annoying that sometimes I have to change my plans because of my dog.**

| Strongly disagree | Disagree | Somewhat disagree | Neither agree nor disagree | Somewhat agree | Agree | Strongly agree |
|---|---|---|---|---|---|---|
| ☐ | ☐ | ☐ | ☐ | ☐ | ☐ | ☐ |

10. **How often do you cuddle your dog?**

| Never | Almost never | Occasionally | More than occasionally | Often | Very often | Extremely often |
|---|---|---|---|---|---|---|
| ☐ | ☐ | ☐ | ☐ | ☐ | ☐ | ☐ |

11. **My dog is constantly attentive to me.**

| Strongly disagree | Disagree | Somewhat disagree | Neither agree nor disagree | Somewhat agree | Agree | Strongly agree |
|---|---|---|---|---|---|---|
| ☐ | ☐ | ☐ | ☐ | ☐ | ☐ | ☐ |

12. **How often do you groom your dog?**

| Never | Almost never | Occasionally | More than occasionally | Often | Very often | Extremely often |
|---|---|---|---|---|---|---|
| ☐ | ☐ | ☐ | ☐ | ☐ | ☐ | ☐ |

13. **My dog costs too much money.**

| Strongly disagree | Disagree | Somewhat disagree | Neither agree nor disagree | Somewhat agree | Agree | Strongly agree |
|---|---|---|---|---|---|---|
| ☐ | ☐ | ☐ | ☐ | ☐ | ☐ | ☐ |

14. **How often do you tell your dog things you do not tell anyone else?**

| Never | Almost Never | Occasionally | More than occasionally | Often | Very often | Extremely often |
|---|---|---|---|---|---|---|
| ☐ | ☐ | ☐ | ☐ | ☐ | ☐ | ☐ |

15. **How often do you feel that looking after your dog is a chore?**

| Never | Almost Never | Occasionally | More than occasionally | Often | Very often | Extremely often |
|---|---|---|---|---|---|---|
| ☐ | ☐ | ☐ | ☐ | ☐ | ☐ | ☐ |

16. **How traumatic do you think it will be for you when your dog dies?**

| Not at all traumatic | A little traumatic | Somewhat traumatic | Quite traumatic | Very traumatic | Extremely traumatic | Unbearably traumatic |
|---|---|---|---|---|---|---|
| ☐ | ☐ | ☐ | ☐ | ☐ | ☐ | ☐ |

17. **How often do you pet your dog?**

| Never | Almost Never | Occasionally | More than occasionally | Often | Very often | Extremely often |
|---|---|---|---|---|---|---|
| ☐ | ☐ | ☐ | ☐ | ☐ | ☐ | ☐ |

18. **If everyone else left me, my dog would still be there for me.**

| Strongly disagree | Disagree | Somewhat disagree | Neither agree nor disagree | Somewhat agree | Agree | Strongly agree |
|---|---|---|---|---|---|---|
| ☐ | ☐ | ☐ | ☐ | ☐ | ☐ | ☐ |

19. **My dog makes too much mess.**

| Strongly disagree | Disagree | Somewhat disagree | Neither agree nor disagree | Somewhat agree | Agree | Strongly agree |
|---|---|---|---|---|---|---|
| ☐ | ☐ | ☐ | ☐ | ☐ | ☐ | ☐ |

20. **How often do you play games with your dog?**

| Never | Almost Never | Occasionally | More than occasionally | Often | Very often | Extremely often |
|---|---|---|---|---|---|---|
| ☐ | ☐ | ☐ | ☐ | ☐ | ☐ | ☐ |

21. **How often do you feel that having your dog is more trouble than it is worth?**

| Never | Almost Never | Occasionally | More than occasionally | Often | Very often | Extremely often |
|---|---|---|---|---|---|---|
| ☐ | ☐ | ☐ | ☐ | ☐ | ☐ | ☐ |

22. **How often do you talk to your dog?**

| Never | Almost Never | Occasionally | More than occasionally | Often | Very often | Extremely often |
|---|---|---|---|---|---|---|
| ☐ | ☐ | ☐ | ☐ | ☐ | ☐ | ☐ |

23. **My dog helps me get through tough times.**

| Strongly disagree | Disagree | Somewhat disagree | Neither agree nor disagree | Somewhat agree | Agree | Strongly agree |
|---|---|---|---|---|---|---|
| ☐ | ☐ | ☐ | ☐ | ☐ | ☐ | ☐ |

24. **How often do you buy your dog gifts?**

| Never | Almost never | Occasionally | More than occasionally | Often | Very often | Extremely often |
|---|---|---|---|---|---|---|
| ☐ | ☐ | ☐ | ☐ | ☐ | ☐ | ☐ |

25. **How difficult is it to look after your dog?**

| Very easy | Easy | Somewhat easy | Neither difficult nor easy | Somewhat difficult | Difficult | Very difficult |
|---|---|---|---|---|---|---|
| ☐ | ☐ | ☐ | ☐ | ☐ | ☐ | ☐ |

26. **How often do you spend time enjoying watching your dog?**

| Never | Almost never | Occasionally | More than occasionally | Often | Very often | Extremely often |
|---|---|---|---|---|---|---|
| ☐ | ☐ | ☐ | ☐ | ☐ | ☐ | ☐ |

27. **There are major aspects of owning a dog I do not like.**

| Strongly disagree | Disagree | Somewhat disagree | Neither agree nor disagree | Somewhat agree | Agree | Strongly agree |
|---|---|---|---|---|---|---|
| ☐ | ☐ | ☐ | ☐ | ☐ | ☐ | ☐ |

28. **How often do you give your dog food treats?**

| Never | Almost never | Occasionally | More than occasionally | Often | Very often | Extremely often |
|---|---|---|---|---|---|---|
| ☐ | ☐ | ☐ | ☐ | ☐ | ☐ | ☐ |

**Administration and Scoring Instructions for the DORS28 and DORS12**

There are two versions of the Dog Owner Relationship Scale (DORS), a 28-item version and a 12-item version. The 28-item scale has good psychometric properties and can be used to generate five variables – an overall measure of the relationship containing all 28 items, and four subscale scores, each of which measures a different aspect of the relationship: Perceived Costs, Affectionate Engagement, Emotional Reliance and Active Engagement.

Use of the 28-item version is strongly recommended unless brevity is required, in which case good approximations of all measures can be obtained by administering only the first three items in each subscale, twelve items in total (DORS12). When administered online, the scale can be personalized by replacing the words 'your dog' and 'my dog' with the dog's name.

Each item is scored on a seven-point scale, from 1 to 7. As specified below, items in the Perceived Costs subscale should be reverse-scored, such that a higher score indicates fewer perceived costs.

**To administer and score the DORS28**

Administer all items.

To calculate the Perceived Costs subscale score (DORS28-PCO), reverse score items 1, 5, 9, 13, 15, 19, 21, 25 and 27. Then add the scores and divide by 9.

To calculate the Affectionate Engagement subscale score (DORS28-AFF), add the scores for items 2, 6, 10, 14, 16, 17, 22, 26 and 28. Then divide by 9.

To calculate the Emotional Reliance subscale score (DORS28-EMR), add the scores for items 3, 7, 11, 18 and 23. Then divide by 5.

To calculate the Active Engagement subscale score (DORS28-ENG), add the scores for items 4, 8, 12, 20 and 24. Then divide by 5.

To calculate the DORS28 total score (DORS28-TOT), sum the four subscale scores.

The range for each subscale is 1-7. For the total score, the range is 4-28.

**To administer and score the DORS12**

Administer only items 1 through 12.

To calculate the Perceived Costs subscale score (DORS12-PCO), reverse score items 1, 5 and 9. Then add the scores and divide by 3.

To calculate the Affectionate Engagement subscale score (DORS12-AFF), add the scores for items 2, 6 and 10, then divide by 3.

To calculate the Emotional Reliance subscale score (DORS12-EMR), add the scores for items 3, 7 and 11, then divide by 3.

To calculate the Active Engagement subscale score (DORS12-ENG), add the scores for items 4, 8 and 12, then divide by 3.

To calculate the DORS12 total score (DORS12-TOT), add the four subscale scores.

The range for each subscale is 1-7. For the total score, the range is 4-28.
