# Supplementary material for: New 28-Item and 12-Item Dog Owner Relationship Scales: Contemporary Versions of the MDORS with a Revised Four-Component Structure
Source: Animals (Basel). 2025 Feb 21;15(5):632. doi: 10.3390/ani15050632 (PMC11898123; doi:10.3390/ani15050632)
Supplement: Supplementary file 1 [file animals-15-00632-s001.zip › File S1 - Survey.pdf]

File S1: Survey

Participant Information and Consent Form

The research is being carried out by the following researchers:
Prof Pauleen Bennett, Dr Tiffani Howell, Dr Vanessa Rohlf and Ms Deanna Tepper, School of Psychology and Public Health, La Trobe University

Chief Investigator: Prof Pauleen Bennett
Associate Investigators: Dr Tiffani Howell, Dr Vanessa Rohlf and Ms Deanna Tepper
This research is supported by in kind support by La Trobe University.

1. What is the study about?
You are invited to participate in a study about the nature of dog-owner relationships.

2. Do I have to participate?
Being part of this study is voluntary. If you want to be part of the study, we ask that you read the information below carefully. You can read the information below and decide at the end if you do not want to participate. If you decide not to participate this won't affect your relationship with La Trobe University.

3. Who is being asked to participate?

You have been asked to participate because you are registered with Prolific and:

- are at least 18 years old
- have a pet dog
- can read and write in English, the language of the survey
- live in a country from which we are collecting data

4. What will I be asked to do?
If you want to take part in this study, we will ask you to complete a brief (5-8 minute) survey about your relationship with your dog. Some of the questions are very similar, so please read the questions and their response options very carefully.

5. What are the benefits?
You will receive a small payment from Prolific for completing the survey. The expected benefits to society in general are to improve our understanding of the relationship between pets and their owners. By developing a more thorough understanding of this relationship, we may be able to create targeted campaigns to improve relationship quality over time, benefitting pets and their owners.

6. What are the risks?
With any study there are (1) risks we know about, (2) risks we don't know about, and (3) risks we don't expect. We do not foresee any risks associated with this study, but if you experience something that you aren't sure about, please contact us immediately so we can discuss the best way to manage your concerns:

Pauleen Bennett, Professor

Tel: +61 3 5444 7460

Email: pauleen.bennett@latrobe.edu.au

7. What will happen to information about me?
By clicking on the 'I agree, start questionnaire' button, this tells us you want to take part in the study.

We will collect, store, and publish information about you in ways that will not reveal who you are. We will keep your information for 5 years after the project is completed. After this time, we will destroy all of your data. The storage, transfer and destruction of your data will be undertaken in accordance with the Research Data Management Policy https://policies.latrobe.edu.au/document/view.php?id=106/.

8. Will I hear about the results of the study?

We will let you know about the results of the study by sending you an emailed summary if you request one, sometime after November 2024. To receive a copy of the results, please email the lead investigator, Pauleen Bennett, at Pauleen.bennett@latrobe.edu.au

9. What if I change my mind?

If you change your mind after beginning the survey, we cannot withdraw your responses because we cannot link who you are with your questionnaire responses.

We will not know whether you personally have decided to withdraw from the survey, and your decision to withdraw at any point will not affect your relationship with La Trobe University

10. Who can I contact for questions or want more information?
If you would like to speak to us, please use the contact details below:
Pauleen Bennett, Professor

Tel: +61 3 5444 7460

Email: pauleen.bennett@latrobe.edu.au

11. What if I have a complaint?
If you have a complaint about any part of this study, please contact:
Senior Research Ethics Officer

Tel: +61 3 9479 1443

Email: humanethics@latrobe.edu.au

Quote Ethics Reference Number: EIBX24011

To download a copy of this information statement, click on the link below

[Attachment: "PICF DORS Oct 2024.pdf"]

---

1) Participant declaration
I (the participant) have read and understood the Participant Information Statement. I agree to participate in the study. I know I can withdraw at any time by navigating away from the website. I agree information provided by me or with my permission during the project may be included in a thesis, presentation and published in journals on the condition that I cannot be identified.

○ I agree, start questionnaire

Demographics

Please complete the survey below.Thank you!

What is your Prolific ID?	__________________________________
Do you own a dog?	◯ Yes<br>◯ No
How many dogs do you have?	◯ 1<br>◯ 2<br>◯ 3<br>◯ 4<br>◯ 5<br>◯ 6 or more
What other kinds of pets do you have? Please check all that apply	☐ Cat<br>☐ Other
Please specify other	__________________________________

In what year were you born?

- ◯ 1920
- ◯ 1921
- ◯ 1922
- ◯ 1923
- ◯ 1924
- ◯ 1925
- ◯ 1926
- ◯ 1927
- ◯ 1928
- ◯ 1929
- ◯ 1930
- ◯ 1931
- ◯ 1932
- ◯ 1933
- ◯ 1934
- ◯ 1935
- ◯ 1936
- ◯ 1937
- ◯ 1938
- ◯ 1939
- ◯ 1940
- ◯ 1941
- ◯ 1942
- ◯ 1943
- ◯ 1944
- ◯ 1945
- ◯ 1946
- ◯ 1947
- ◯ 1948
- ◯ 1949
- ◯ 1950
- ◯ 1951
- ◯ 1952
- ◯ 1953
- ◯ 1954
- ◯ 1955
- ◯ 1956
- ◯ 1957
- ◯ 1958
- ◯ 1959
- ◯ 1960
- ◯ 1961
- ◯ 1962
- ◯ 1963
- ◯ 1964
- ◯ 1965
- ◯ 1966
- ◯ 1967
- ◯ 1968
- ◯ 1969
- ◯ 1970
- ◯ 1971
- ◯ 1972
- ◯ 1973
- ◯ 1974
- ◯ 1975
- ◯ 1976
- ◯ 1977
- ◯ 1978
- ◯ 1979
- ◯ 1980
- ◯ 1981
- ◯ 1982
- ◯ 1983
- ◯ 1984
- ◯ 1985
- ◯ 1986
- ◯ 1987
- ◯ 1988

	◯ 1989<br>◯ 1990<br>◯ 1991<br>◯ 1992<br>◯ 1993<br>◯ 1994<br>◯ 1995<br>◯ 1996<br>◯ 1997<br>◯ 1998<br>◯ 1999<br>◯ 2000<br>◯ 2001<br>◯ 2002<br>◯ 2003<br>◯ 2004<br>◯ 2005<br>◯ 2006<br>◯ 2007
What is your gender?	◯ Male<br>◯ Female<br>◯ I identify my gender as... (please describe below)<br>◯ Prefer not to say
Please specify how you describe your gender	______________________
What is the highest level of education you have completed?	◯ No formal schooling<br>◯ Year/Grade 10 or below (up to age 16 years)<br>◯ Year/Grade 11 or 12 (above age 16 years)<br>◯ Certificate, diploma, advanced diploma, associate degree, technical/trade qualification, TAFE<br>◯ University/College degree (Bachelor's degree)<br>◯ University/College degree (Master's, PhD, or equivalent)<br>◯ Other<br>◯ Prefer not to say
Please describe other	______________________
Which of the following best describes the area in which your home is located?	◯ Urban (inner city)<br>◯ Suburban (over 10km/6mi from inner city)<br>◯ Regional city (population 50,000 or more)<br>◯ Country town (population 50,000 or less)<br>◯ Rural
What kind of dwelling is your home?	◯ House<br>◯ Semi-detached, terrace house, townhouse<br>◯ Flat, unit, apartment<br>◯ Other
Please describe other	______________________
Does the dwelling include the following?	◯ Large outside space (farm, acreage)<br>◯ Medium outside space (large house yard, small acreage)<br>◯ Small outside space (small yard, patio, balcony)<br>◯ No outside space

Which of the following best describes your current situation in relation to paid work?	◯ Retired<br>◯ Unemployed<br>◯ Unable to work<br>◯ Engaged in home duties<br>◯ Part time/casual paid work (30 hours or less per week)<br>◯ Full time paid work (more than 30 hours per week)<br>◯ Student<br>◯ Other<br>◯ I'd rather not say
Please describe other	__________________________________
In total, including yourself, how many adults aged 18 or over live in your household most of the time?	◯ 1<br>◯ 2<br>◯ 3<br>◯ 4 or more
How many children aged 17 years or younger live in your household most of the time?	◯ 0<br>◯ 1<br>◯ 2<br>◯ 3<br>◯ 4 or more
Please indicate the age(s) of the child/children in your home. Please select all that apply.	☐ 0 to 4 years<br>☐ 5 to 8 years<br>☐ 9 to 12 years<br>☐ 13 to 17 years
Which of these best describes your current relationship status?	◯ Single/Never married<br>◯ De-facto/common law or living together<br>◯ Married<br>◯ Separated, divorced, or widowed<br>◯ I'd rather not say
When did you own or live with your first dog?	◯ As a child (12 years old or younger)<br>◯ As an adolescent (13 to 17 years old)<br>◯ As an adult (18 years or older)
How many dogs have you had before the current one, including any that you lived with as a child?	◯ None<br>◯ 1<br>◯ 2<br>◯ 3<br>◯ 4 or more

Pet demographics

Please complete the survey below.Thank you!

Now we'd like you to answer some questions about your pet dog. If you have more than one pet dog, please select the dog with the name that starts with the letter closest to 'A'.

What is this dog's name?

____

How old is [petname]?

- ◯ Less than 6 months
- ◯ 6-12 months
- ◯ 1 to 2 years
- ◯ 3 to 8 years
- ◯ 9 to 12 years
- ◯ 13 years or older
- ◯ Don't know

How old was [petname] when you got him/her?

- ◯ Less than 1 month
- ◯ 1 to 4 months
- ◯ 5 to 12 months
- ◯ 1 to 3 years
- ◯ Over 3 years
- ◯ Don't know

Is [petname] sterilized (spayed/neutered/desexed) or entire?

- ◯ Male - entire/intact
- ◯ Male - desexed/neutered/sterilized
- ◯ Female - entire/intact
- ◯ Female - desexed/spayed/sterilized
- ◯ Don't know

What breed or type is [petname]?

____

Approximately how much does [petname] weigh? Please write only numbers

____

Did you write [petname]'s weight in pounds (lbs) or kilograms (kg)?

- ◯ pounds (lbs)
- ◯ kilograms (kg)

Where did you get [petname]?

- ◯ Bred him/her myself
- ◯ From a friend/family member/acquaintance
- ◯ From a breeder
- ◯ From a shelter or rescue service
- ◯ I found him/her
- ◯ Gift
- ◯ Inherited
- ◯ Pet shop
- ◯ Veterinarian
- ◯ Other

Please describe other

____

Where does [petname] spend most of his/her waking time?

- ◯ Inside the house
- ◯ Outside the house
- ◯ Both inside and outside the house equally
- ◯ Other

Please describe other	__________________________________
How satisfied are you with [petname]'s behaviour?	◯ Very dissatisfied<br>◯ Dissatisfied<br>◯ Neither satisfied nor dissatisfied<br>◯ Satisfied<br>◯ Very satisfied
Please explain your answer	__________________________________
How satisfied are you with [petname]'s health?	◯ Very dissatisfied<br>◯ Dissatisfied<br>◯ Neither satisfied nor dissatisfied<br>◯ Satisfied<br>◯ Very satisfied
Please explain your answer	__________________________________
What sorts of activities do you do with [petname]? Please select all that apply.	☐ Sports (e.g., agility, flyball)<br>☐ Scent tracking<br>☐ Obedience training<br>☐ Search and rescue<br>☐ Animal-assisted interventions (e.g., hospital visitation with dog)<br>☐ Trick training<br>☐ General pet activities (e.g. grooming, play, lead walks)<br>☐ Other<br>☐ None
Please describe other	__________________________________
Relative to other dog owners, which of the following best describes the quality of your relationship with [petname]?	◯ Much worse<br>◯ Somewhat worse<br>◯ Slightly worse<br>◯ About the same<br>◯ Slightly better<br>◯ Somewhat better<br>◯ Much better

C/DORS

Please complete the survey below.Thank you!

Please consider each of the following statements and indicate which option most describes how you feel or act. We are interested in your opinions. There are no correct or incorrect responses.
Some of these questions are very similar, but the response options may vary. Please read each question and their response options carefully before answering, and don't worry if you see some questions that look a lot alike.

1) How difficult is it to look after [petname]?
- ◯ Very easy
- ◯ Easy
- ◯ Somewhat easy
- ◯ Neither difficult nor easy
- ◯ Somewhat difficult
- ◯ Difficult
- ◯ Very difficult

2) How often do you play games with [petname]?
- ◯ Never
- ◯ Almost never
- ◯ Occasionally
- ◯ More than occasionally
- ◯ Often
- ◯ Very often
- ◯ Extremely often

3) [petname] gives me a reason to get up in the morning.
- ◯ Strongly disagree
- ◯ Disagree
- ◯ Somewhat disagree
- ◯ Neither agree nor disagree
- ◯ Somewhat agree
- ◯ Agree
- ◯ Strongly agree

4) There are major aspects of owning [petname] I don't like.
- ◯ Strongly disagree
- ◯ Disagree
- ◯ Somewhat disagree
- ◯ Neither agree nor disagree
- ◯ Somewhat agree
- ◯ Agree
- ◯ Strongly agree

5) How often do you kiss [petname]?
- ◯ Once a month or less
- ◯ About once a week
- ◯ A few times a week
- ◯ About once a day
- ◯ More than once a day
- ◯ About once an hour
- ◯ More than once an hour

6) How often do you tell [petname] things you do not tell anyone else?
- ◯ Never
- ◯ Almost never
- ◯ Occasionally
- ◯ More than occasionally
- ◯ Often
- ◯ Very often
- ◯ Extremely often

7)	I wish [petname] and I never had to be apart	◯ Strongly disagree<br>◯ Disagree<br>◯ Somewhat disagree<br>◯ Neither agree nor disagree<br>◯ Somewhat agree<br>◯ Agree<br>◯ Strongly agree
8)	How often do you cuddle [petname]?	◯ Never<br>◯ Almost never<br>◯ Occasionally<br>◯ More than occasionally<br>◯ Often<br>◯ Very often<br>◯ Extremely often
9)	[petname] makes too much mess.	◯ Strongly disagree<br>◯ Disagree<br>◯ Somewhat disagree<br>◯ Neither agree nor disagree<br>◯ Somewhat agree<br>◯ Agree<br>◯ Strongly agree
10)	How often do you play games with [petname]?	◯ Once a month or less<br>◯ About once a week<br>◯ A few times a week<br>◯ About once a day<br>◯ More than once a day<br>◯ About once an hour<br>◯ More than once an hour
11)	How often do you kiss [petname]?	◯ Never<br>◯ Almost never<br>◯ Occasionally<br>◯ More than occasionally<br>◯ Often<br>◯ Very often<br>◯ Extremely often
12)	It bothers me that [petname] stops me doing things I enjoyed before I owned him/her.	◯ Strongly disagree<br>◯ Disagree<br>◯ Somewhat disagree<br>◯ Neither agree nor disagree<br>◯ Somewhat agree<br>◯ Agree<br>◯ Strongly agree
13)	How often do you spend time enjoying watching [petname]?	◯ Once a month or less<br>◯ About once a week<br>◯ A few times a week<br>◯ About once a day<br>◯ More than once a day<br>◯ About once an hour<br>◯ More than once an hour

14) How often do you talk to [petname]?

- ◯ Never
- ◯ Almost never
- ◯ Occasionally
- ◯ More than occasionally
- ◯ Often
- ◯ Very often
- ◯ Extremely often

15) It is annoying that sometimes I have to change my plans because of [petname]

- ◯ Strongly disagree
- ◯ Disagree
- ◯ Somewhat disagree
- ◯ Neither agree nor disagree
- ◯ Somewhat agree
- ◯ Agree
- ◯ Strongly agree

16) How often do you feel that having [petname] is more trouble than it's worth?

- ◯ Never
- ◯ Almost never
- ◯ Occasionally
- ◯ More than occasionally
- ◯ Often
- ◯ Very often
- ◯ Extremely often

17) [petname] costs too much money

- ◯ Strongly disagree
- ◯ Disagree
- ◯ Somewhat disagree
- ◯ Neither agree nor disagree
- ◯ Somewhat agree
- ◯ Agree
- ◯ Strongly agree

18) How often do you buy [petname] gifts?

- ◯ Once a year or less
- ◯ A few times a year
- ◯ About once a month
- ◯ About once a week
- ◯ A few times a week
- ◯ About once a day
- ◯ More than once a day

19) How often do you pet [petname]?

- ◯ Never
- ◯ Almost never
- ◯ Occasionally
- ◯ More than occasionally
- ◯ Often
- ◯ Very often
- ◯ Extremely often

20) How often do you tell [petname] things you don't tell anyone else?

- ◯ Once a year or less
- ◯ A few times a year
- ◯ About once a month
- ◯ About once a week
- ◯ A few times a week
- ◯ About once a day
- ◯ More than once a day

21) How often do you take [petname] to visit people?
- ◯ Never
- ◯ Almost never
- ◯ Occasionally
- ◯ More than occasionally
- ◯ Often
- ◯ Very often
- ◯ Extremely often

22) How often do you give [petname] food treats?
- ◯ Never
- ◯ Almost never
- ◯ Occasionally
- ◯ More than occasionally
- ◯ Often
- ◯ Very often
- ◯ Extremely often

23) How often do you feel that looking after [petname] is a chore?
- ◯ Once a month or less
- ◯ About once a week
- ◯ A few times a week
- ◯ About once a day
- ◯ More than once a day
- ◯ About once an hour
- ◯ More than once an hour

24) How often do you talk to [petname]?
- ◯ Once a month or less
- ◯ About once a week
- ◯ A few times a week
- ◯ About once a day
- ◯ More than once a day
- ◯ About once an hour
- ◯ More than once an hour

25) How often do you hug [petname]?
- ◯ Never
- ◯ Almost never
- ◯ Occasionally
- ◯ More than occasionally
- ◯ Often
- ◯ Very often
- ◯ Extremely often

26) How often does [petname] stop you doing things you want to?
- ◯ Once a month or less
- ◯ About once a week
- ◯ A few times a week
- ◯ About once a day
- ◯ More than once a day
- ◯ About once an hour
- ◯ More than once an hour

27) I would like to have [petname] near me all the time
- ◯ Strongly disagree
- ◯ Disagree
- ◯ Somewhat disagree
- ◯ Neither agree nor disagree
- ◯ Somewhat agree
- ◯ Agree
- ◯ Strongly agree

28) How often do you enjoy spending time watching [petname]?

- ◯ Never
- ◯ Almost never
- ◯ Occasionally
- ◯ More than occasionally
- ◯ Often
- ◯ Very often
- ◯ Extremely often

29) If everyone else left me, [petname] would still be there for me.

- ◯ Strongly disagree
- ◯ Disagree
- ◯ Somewhat disagree
- ◯ Neither agree nor disagree
- ◯ Somewhat agree
- ◯ Agree
- ◯ Strongly agree

30) How often do you feel that having [petname] is more trouble than it's worth?

- ◯ Once a month or less
- ◯ About once a week
- ◯ A few times a week
- ◯ About once a day
- ◯ More than once a day
- ◯ About once an hour
- ◯ More than once an hour

31) [petname] helps me get through tough times

- ◯ Strongly disagree
- ◯ Disagree
- ◯ Somewhat disagree
- ◯ Neither agree nor disagree
- ◯ Somewhat agree
- ◯ Agree
- ◯ Strongly agree

32) How often do you cuddle [petname]?

- ◯ Once a month or less
- ◯ About once a week
- ◯ A few times a week
- ◯ About once a day
- ◯ More than once a day
- ◯ About once an hour
- ◯ More than once an hour

33) How often do you feel that looking after [petname] is a chore?

- ◯ Never
- ◯ Almost never
- ◯ Occasionally
- ◯ More than occasionally
- ◯ Often
- ◯ Very often
- ◯ Extremely often

34) How often do you take [petname] in the car, on your bike, or on public transport?

- ◯ Never
- ◯ Almost never
- ◯ Occasionally
- ◯ More than occasionally
- ◯ Often
- ◯ Very often
- ◯ Extremely often

35) [petname] provides me with constant companionship.
- ◯ Strongly disagree
- ◯ Disagree
- ◯ Somewhat disagree
- ◯ Neither agree nor disagree
- ◯ Somewhat agree
- ◯ Agree
- ◯ Strongly agree

36) How often do you have [petname] with you while relaxing, e.g., watching TV?
- ◯ Once a month or less
- ◯ About once a week
- ◯ A few times a week
- ◯ About once a day
- ◯ More than once a day
- ◯ About once an hour
- ◯ More than once an hour

37) How often do you buy [petname] gifts?
- ◯ Never
- ◯ Almost never
- ◯ Occasionally
- ◯ More than occasionally
- ◯ Often
- ◯ Very often
- ◯ Extremely often

38) [petname] is there whenever I need to be comforted.
- ◯ Strongly disagree
- ◯ Disagree
- ◯ Somewhat disagree
- ◯ Neither agree nor disagree
- ◯ Somewhat agree
- ◯ Agree
- ◯ Strongly agree

39) How traumatic do you think it will be for you when [petname] dies?
- ◯ Not at all traumatic
- ◯ A little traumatic
- ◯ Somewhat traumatic
- ◯ Quite traumatic
- ◯ Very traumatic
- ◯ Extremely traumatic
- ◯ Unbearably traumatic

40) How often do you groom [petname]?
- ◯ Never
- ◯ Almost never
- ◯ Occasionally
- ◯ More than occasionally
- ◯ Often
- ◯ Very often
- ◯ Extremely often

41) How often do you pet [petname]?
- ◯ Once a month or less
- ◯ About once a week
- ◯ A few times a week
- ◯ About once a day
- ◯ More than once a day
- ◯ About once an hour
- ◯ More than once an hour

42) How often do you take [petname] to visit people?

- ◯ Once a year or less
- ◯ A few times a year
- ◯ About once a month
- ◯ About once a week
- ◯ A few times a week
- ◯ About once a day
- ◯ More than once a day

43) How often do you give [petname] food treats?

- ◯ Once a month or less
- ◯ About once a week
- ◯ A few times a week
- ◯ About once a day
- ◯ More than once a day
- ◯ About once an hour
- ◯ More than once an hour

44) How often do you take [petname] in the car, on your bike, or on public transport?

- ◯ Once a year or less
- ◯ A few times a year
- ◯ About once a month
- ◯ About once a week
- ◯ A few times a week
- ◯ About once a day
- ◯ More than once a day

45) How often does [petname] stop you doing things you want to?

- ◯ Never
- ◯ Almost never
- ◯ Occasionally
- ◯ More than occasionally
- ◯ Often
- ◯ Very often
- ◯ Extremely often

46) How often do you hug [petname]?

- ◯ Once a month or less
- ◯ About once a week
- ◯ A few times a week
- ◯ About once a day
- ◯ More than once a day
- ◯ About once an hour
- ◯ More than once an hour

47) How often do you have [petname] with you while relaxing, e.g., watching TV?

- ◯ Never
- ◯ Almost never
- ◯ Occasionally
- ◯ More than occasionally
- ◯ Often
- ◯ Very often
- ◯ Extremely often

48) How often do you groom [petname]?

- ◯ Once a year or less
- ◯ A few times a year
- ◯ About once a month
- ◯ About once a week
- ◯ A few times a week
- ◯ About once a day
- ◯ More than once a day

49) [petname] is constantly attentive to me.

◯ Strongly disagree
◯ Disagree
◯ Somewhat disagree
◯ Neither agree nor disagree
◯ Somewhat agree
◯ Agree
◯ Strongly agree

LAPS

Please complete the survey below.Thank you!

We'd like to ask you whether you agree or disagree with some very brief statements about [petname]. For each statement, please tell us whether you strongly agree, somewhat agree, somewhat disagree, or strongly disagree.

		Strongly disagree	Somewhat disagree	Somewhat agree	Strongly agree
1)	[petname] means more to me than any of my friends.	◯	◯	◯	◯
2)	Quite often I confide in [petname].	◯	◯	◯	◯
3)	I believe that pets should have the same rights and privileges as family members.	◯	◯	◯	◯
4)	I believe [petname] is my best friend.	◯	◯	◯	◯
5)	Quite often, my feelings toward people are affected by the way they react to [petname].	◯	◯	◯	◯
6)	I love [petname] because he/she is more loyal to me than most of the people in my life.	◯	◯	◯	◯
7)	I enjoy showing other people pictures of [petname].	◯	◯	◯	◯
8)	I think [petname] is just a pet.	◯	◯	◯	◯
9)	I love [petname] because he/she never judges me.	◯	◯	◯	◯
10)	[petname] knows when I'm feeling bad.	◯	◯	◯	◯
11)	I often talk to other people about [petname].	◯	◯	◯	◯
12)	[petname] understands me.	◯	◯	◯	◯
13)	I believe that loving [petname] helps me stay healthy.	◯	◯	◯	◯
14)	Pets deserve as much respect as humans do.	◯	◯	◯	◯
15)	[petname] and I have a very close relationship.	◯	◯	◯	◯
16)	I would do almost anything to take care of [petname].	◯	◯	◯	◯
17)	I play with [petname] quite often.	◯	◯	◯	◯
18)					

I consider [petname] to be a great companion.	◯	◯	◯	◯
19) [petname] makes me feel happy.	◯	◯	◯	◯
20) I feel that [petname] is a part of my family.	◯	◯	◯	◯
21) I am not very attached to [petname].	◯	◯	◯	◯
22) Owning a pet adds to my happiness.	◯	◯	◯	◯
23) I consider [petname] to be a friend.	◯	◯	◯	◯

Final questions

How bonded do you think [petname] is to you?	◯ Not at all<br>◯ Very little<br>◯ A little<br>◯ Somewhat<br>◯ Quite a bit<br>◯ Very<br>◯ Extremely
Please write three adjectives that best represent your relationship with [petname]	__________________________________
Please write three adjectives that best represent [petname]	__________________________________
